# Supplementary material for: CD38-mediated metabolic reprogramming promotes the stability and suppressive function of regulatory T cells in tumor
Source: Sci Adv. 2025 Mar 21;11(12):eadt2117. doi: 10.1126/sciadv.adt2117 (PMC11927613; doi:10.1126/sciadv.adt2117)
Supplement: Supplementary file 2 [file sciadv.adt2117.v1.pdf]

IMMUNOLOGY

CD38-mediated metabolic reprogramming promotes the stability and suppressive function of regulatory T cells in tumor

Copyright © 2025 The Authors, some rights reserved; exclusive licensee American Association for the Advancement of Science. No claim to original U.S. Government Works. Distributed under a Creative Commons Attribution License 4.0 (CC BY).

Ishita Sarkar[1,2], Debashree Basak[1,2], Puspendu Ghosh[1,2], Anupam Gautam[3,4], Arpita Bhoumik[1], Praveen Singh[2,5], Anwesha Kar[1,2], Shaun Mahanti[1,2], Snehanshu Chowdhury[1], Lagnajita Chakraborty[2,6], Soumya Mondal[7], Ramanuj Mukherjee[8]†, Shikhar Mehrotra[9], Saikat Majumder[2,6], Shantanu Sengupta[2,5], Sandip Paul[10], Shilpak Chatterjee[1,2]*

In the tumor microenvironment (TME), regulatory T cells (T_{regs}) adapt their metabolism to thrive in low-glucose, high-lactate conditions, but the mechanisms remain unclear. Our study identifies CD38 as a key regulator of this adaptation by depleting nicotinamide adenine dinucleotide (oxidized form) (NAD^+), redirecting lactate-derived pyruvate toward phosphoenolpyruvate and bypassing the tricarboxylic acid (TCA) cycle. This prevents accumulation of α-ketoglutarate, which destabilizes T_{regs} by inducing hypermethylation at the *Foxp3* locus. Restoring NAD^+ with nicotinamide mononucleotide reverses this adaptation, pushing T_{regs} back to the TCA cycle and reducing their suppressive function. In YUMM1.7 melanoma-bearing mice, small-molecule CD38 inhibition selectively destabilizes intratumoral T_{regs}, sparking robust antitumor immunity. These findings reveal that targeting the CD38-NAD^+ axis disrupts T_{regs} metabolic adaptation and offers a strategy to enhance antitumor responses.

INTRODUCTION

Regulatory T cells (T_{regs}) are crucial for maintaining peripheral tolerance but pose a major challenge in cancer immunotherapy (*1*, *2*). In the tumor microenvironment (TME), T_{regs} use various suppressive mechanisms to hinder T cell functions essential for tumor control (*3*, *4*). T_{regs} have been shown to promote hyper-progression of cancer after programmed cell death protein 1 (PD1) therapy in a fraction of patients with gastric cancer due to the amplification of $PD1^+$ T_{regs}, which are highly suppressive (*1*). While T_{regs}-depleting approaches lead to bolstering the effector functions of T cells and have generated promising antitumor response, the clinical implication is limited because of the development of severe autoimmunity caused by systemic loss of T_{regs} (*5*). Therefore, identifying strategies to selectively target intratumoral T_{regs} is paramount for successful cancer immunotherapy.

The metabolic adaptability of T cells, enabling cells to adjust and switch between different metabolic pathways in response to changes in the environment or functional requirements, is imperative to evoke the tissue-specific immune response (*6*). Studies have revealed that, within the complex and often hostile TME, T_{regs} undergo specific metabolic adaptations to support their survival and function (*7*–*11*). It has been shown that, while a low-glucose, high-lactate TME impairs the functionality of effector T cells (T_{effs}), T_{regs} adjust their metabolic machinery and nutrient-sensing pathways to use lactate, meeting their bioenergetic demands (*7*, *8*, *12*, *13*). These adaptations are also found to be crucial for maintaining the cellular identity and suppressive functions of T_{regs} within the tumor (*8*). However, the cellular mechanisms that reprogram the metabolic machinery of T_{regs}, enabling them to adopt metabolic traits that support their functionality and survival in the TME, remain unclear.

Efforts to understand lactate metabolism in intratumoral T_{regs} using $^{13}C_3$ L-lactate have revealed an alternative pathway that promotes phosphoenolpyruvate (PEP) production, indicating a shift toward gluconeogenesis (*8*). PEP, essential for gluconeogenesis, can be derived from oxaloacetate (OAA) either through cytosolic malate dehydrogenase, converting TCA cycle–derived malate to OAA and then to PEP via cytosolic PEP carboxykinase–C (PEPCK-C), or through pyruvate carboxylase (PC) converting mitochondrial pyruvate to OAA, followed by PEP production via mitochondrial PEPCK-M (*14*, *15*). This raises the question of whether intratumoral T_{regs} prefer one pathway over another in lactate utilization to support their metabolic needs. Such a preference could have major consequences, as promoting the TCA cycle might increase the levels of key intermediate metabolites, including α-ketoglutarate (α-KG), impinging on their suppressive potential and stability (*11*, *16*). A recent study has shown that T_{regs} within the TME exhibit reduced mitochondrial mass and respiration compared to splenic T_{regs}, which primarily use oxidative phosphorylation (OXPHOS) to support their suppressive activity (*9*). Moreover, reduced mitochondrial OXPHOS in intratumoral T_{regs} has been identified as critical for their suppressive function (*9*, *11*). These findings suggest that intratumoral T_{regs} in the TME must adapt mechanisms to ensure efficient lactate utilization through the pathway that meets their bioenergetic demands while maintaining stability and functionality. However, the regulatory mechanisms governing this decision-making process in lactate metabolism in T_{regs} remain poorly understood.

CD38, an ectonucleotidase, has emerged as an important metabolic checkpoint in T cells due to its NADase activity, which regulates intracellular nicotinamide adenine dinucleotide (oxidized form)

[1]Division of Cancer Biology and Inflammatory Disorder, IICB-Translational Research Unit of Excellence, CSIR–Indian Institute of Chemical Biology, Kolkata 700032, India. [2]Academy of Scientific and Innovative Research (AcSIR), Ghaziabad 201002, India. [3]Institute for Bioinformatics and Medical Informatics, University of Tübingen, Sand 14, 72076 Tübingen, Germany. [4]International Max Planck Research School "From Molecules to Organisms", Max Planck Institute for Biology Tübingen, Max-Planck-Ring 5, 72076, Tübingen, Germany. [5]CSIR–Institute of Genomics and Integrative Biology, New Delhi 110020, India. [6]Division of Infectious Disease and Immunology, CSIR-Indian Institute of Chemical Biology, Kolkata 700032, India. [7]Department of Urology, IPGME&R and SSKM Hospital, Kolkata, India. [8]R.G. Kar Medical College and Hospitals, Kolkata, India. [9]Medical University of South Carolina. [10]Center for Health Science and Technology, JIS Institute of Advanced Studies and Research, JIS University, Kolkata, India.
*Corresponding author. Email: schatterjee@iicb.res.in
†Present address: Chittaranjan National Cancer Institute, Kolkata, India.

(NAD^+) pools (17–19). Studies have shown that elevated CD38 expression on T_{effs} affects mitochondrial metabolism and, thereby, their functionality at the tumor site (19, 20). Recently, elevated expression of CD38 has been reported in T_{regs} isolated from patients with different malignancies (21, 22). However, the role of CD38 in the metabolic adaptation and regulation of T_{regs} functionality at the tumor site remains incompletely understood. Our findings demonstrate that CD38 expression regulates the stability and suppressive activity of T_{regs} by modulating intracellular NAD^+ levels. CD38-mediated reduction in NAD^+ content in T_{regs} restricts TCA cycle–mediated generation of α-KG, thus preserving the suppressive methylation pattern of the *Foxp3* gene. Our observations reveal that the CD38-NAD^+ axis dictates the lactate flux in T_{regs} by redirecting its utilization toward the generation of PEP rather than fueling the TCA cycle, thereby supporting T_{regs} function at the tumor site.

RESULTS

Intratumoral T_{regs} express CD38 and exhibit distinct transcriptional features

To elucidate the functional relevance of CD38 in intratumoral T_{regs}, we first examined their abundance at the tumor site. Analysis of tumor tissues and paired peripheral blood samples from patients with breast and muscle-invasive bladder cancer (MIBC) revealed that T_{regs} infiltration, marked by $CD4^+$ and $FoxP3^+$ expression, was significantly higher in tumor tissue than in peripheral blood (fig. S1, A and B), as reported (23, 24). However, upon analysis of $CD4^+FoxP3^+$ cells, we noted that the expression of CD38 was more frequent on intratumoral T_{regs} compared to that on T_{regs} from peripheral blood (Fig. 1, A and B).

To confirm this observation, we used various preclinical models in which mice were subcutaneously implanted with B16-F10 melanoma, YUMM1.7 melanoma, or EL-4 thymoma. As anticipated, the abundance of T_{regs} was markedly higher at tumor sites compared to the spleen and draining lymph nodes (DLNs) (fig. S1, C to E). Notably, across all tumor types assessed, CD38-expressing T_{regs} were significantly enriched at tumor sites compared to those at the spleen and DLN (Fig. 1, C to E). Furthermore, $CD38^+$ T_{regs} were scarce in nonlymphoid tissues such as the skin and liver (fig. S1, F to H, and Fig. 1, C to E). When evaluating CD38 expression levels, we found that intratumoral T_{regs} exhibited markedly higher CD38 expression than their lymphoid and nonlymphoid counterparts (Fig. 1, F to H). Together, these findings suggest that intratumoral T_{regs} have a strong propensity to express CD38, in stark contrast to T_{regs} residing in peripheral tissues, where CD38 expression is markedly lower.

Given the recent studies highlighting high heterogeneity among intratumoral T_{regs}, we sought to determine the expression pattern of CD38 in these cells and explore the associated transcriptional signature. To achieve this, we analyzed publicly available single-cell RNA sequencing (scRNA-seq) data of tumor-infiltrating lymphocytes from patients with breast cancer (GSE 114727) (25). After applying stringent quality control and filtering, a total of 17,645 $CD3^+$ T cells were subjected to unsupervised clustering analysis, identifying 12 distinct T cell clusters (fig. S1I). Among these clusters, clusters 1 and 8 were characterized as T_{regs} distinguished by their enriched expression of *CD3G*, *CD4*, and the subset determining master transcription factor *FOXP3* (fig. S1, J and K). To further investigate the transcriptional diversity, we re-clustered 3127 T_{regs} from clusters 1 and 8, revealing five distinct subclusters of intratumoral T_{regs} (Fig. 1I). Clusters 0 and 3, which accounted for 36.65 and 12.98% of total T_{regs}, respectively, were highly enriched in CD38 expression (Fig. 1, J and K). Moreover, genes associated with superior suppressive function and stability in T_{regs}, including *ICOS*, *ID3*, *PIM2*, *TIGIT*, *MCL1*, *IL2RA*, *ISG15*, and *CREM*, were predominant in clusters 0 and 3, which exhibited high CD38 expression (Fig. 1L). Cluster 0 further differed from cluster 3 by expressing additional T cell suppression-associated genes such as *RGS1*, *IFIT1*, and *CRIP1*. In contrast to clusters 0 and 3, the other three clusters (1, 2, and 4) displayed very low CD38 expression. Cluster 2 was characterized as "lymph node–homing T_{regs}" due to their expression of *SELL*, *CCR7*, and *KLF2*. Clusters 1 and 4 remain undefined but were enriched in the expression of *PRMD1*, *FKBP5*, *GPX* (cluster 1), and *BTG2* (cluster 4), genes associated with T_{regs} function and stability (Fig. 1L). Collectively, these data suggest that a substantial fraction of intratumoral T_{regs} expresses CD38 and that CD38-expressing T_{regs} harbor genes that are associated with heightened suppressive function and stability of T_{regs}.

$CD38^{Hi}$ T_{regs} exhibit superior suppressive activity

Having found enriched expression of genes associated with heightened immunosuppression in CD38-expressing T_{regs}, we next interrogated their suppressive potential. Because of the technical challenges in retrieving sufficient $CD38^+$ T_{regs} from the tumor site for functional studies, we first checked whether in vitro–differentiated T_{regs} (iT_{regs}) also expressed CD38. We observed that naïve $CD4^+$ T cells differentiated to T_{eff} barely express CD38, while the frequency of $CD38^+$ cells was significantly higher in iT_{regs}, comprising ~70 to 75% of the iT_{regs} population (Fig. 2A and fig. S2A). Notably, the frequency of $CD38^+$ iT_{regs} could not be further increased upon iT_{regs} differentiation in the tumor-conditioned medium (TME) (fig. S2B) or under hypoxic conditions (fig. S2, C and D), suggesting that transforming growth factor–β (TGF-β) is sufficient to induce CD38 expression on iT_{regs}, as reported earlier (20, 26).

Given the expression of CD38 on iT_{regs}, we examined whether the CD38-expressing iT_{regs} subset also harbors the suppressive gene signature as observed in intratumoral $CD38^+$ T_{regs}. We sorted iT_{regs} based on the CD38 expression and analyzed immunosuppressive gene expression in $CD38^{Hi}$ and $CD38^{Lo}$ subsets. We found that the expression of *Ctla4*, *Icos*, *IL2ra*, *Ikzf2*, *Nrp1*, *Entpd1* (gene encodes CD39), and *Lag3* genes associated with the immunosuppressive potential of T_{regs} was markedly up-regulated in $CD38^{Hi}$ iT_{regs} compared to $CD38^{Lo}$ that in iT_{regs} (Fig. 2B). Next, we subjected the sorted $CD38^{Hi}$ and $CD38^{Lo}$ iT_{regs} to the suppression assay with naïve T cells purified from wild-type (WT) B6 mice. We observed that $CD38^{Hi}$ iT_{regs} were superior to $CD38^{Lo}$ iT_{regs} in suppressing the proliferation and effector cytokine production of T cells at all the tested ratios (Fig. 2, C and D). Notably, this differential suppressive capacity was not due to differences in viability, as both subsets remained equally viable after the assay (fig. S2E). Instead, the superior function of $CD38^{Hi}$ iT_{regs} could partly be attributed to their enhanced retention of FoxP3 expression, as $CD38^{Lo}$ iT_{regs} were more prone to losing FoxP3 expression when cultured for extended periods (Fig. 2, E and F).

To further confirm the role of CD38 in regulating the suppressive potential of iT_{regs}, we differentiated naïve $CD4^+$ T cells from both $CD38^{-/-}$ and WT B6 mice into iT_{regs} and assessed their suppressive potential. We noted that, although there were no overt differences between $CD38^{-/-}$ and WT $CD4^+$ T cells in their differentiation into iT_{regs} (fig. S2F), $CD38^{-/-}$ iT_{regs} were less effective than WT iT_{regs} in

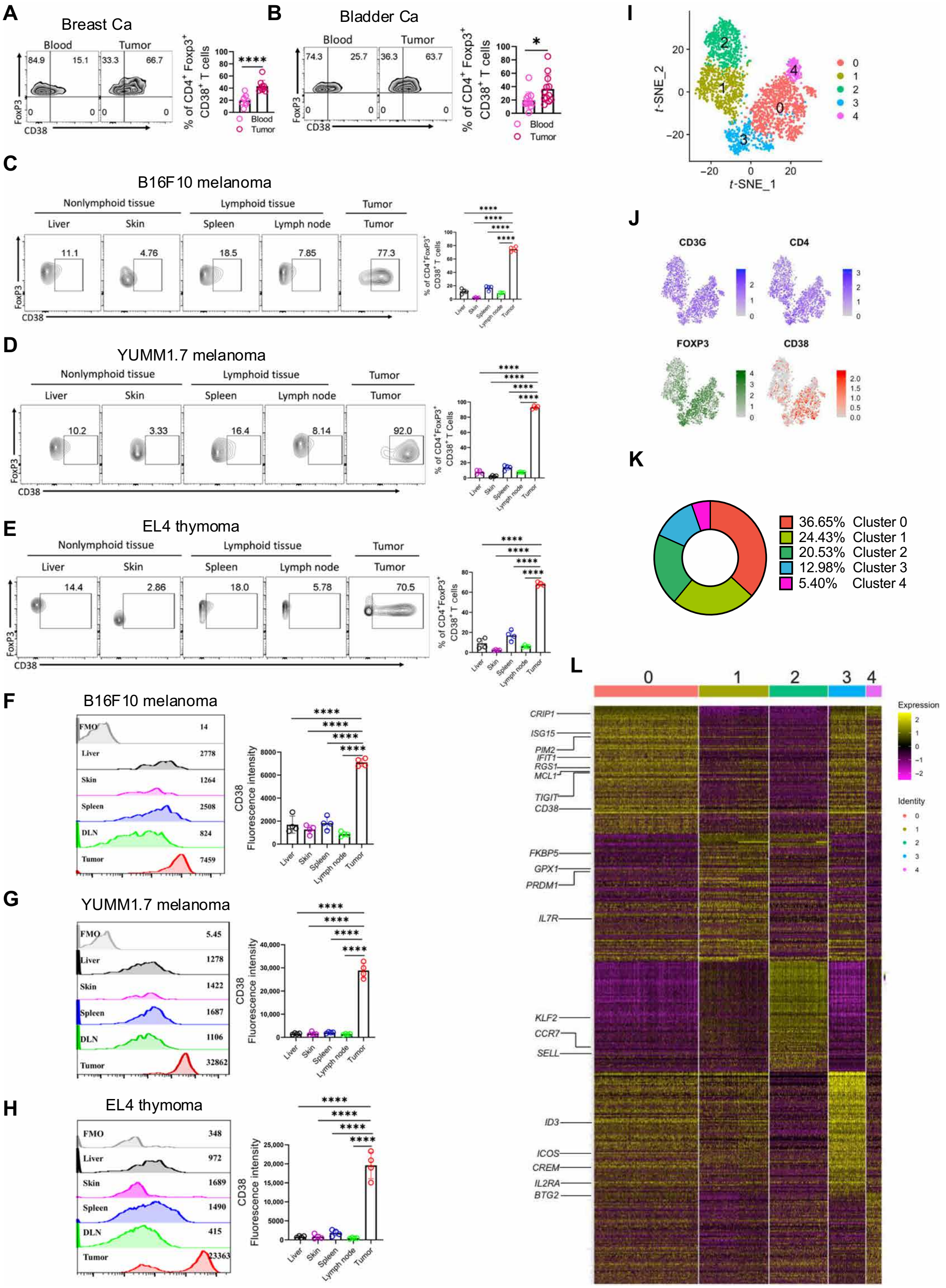

Fig. 1. Intratumoral T_{regs} display elevated CD38 expression. (**A** to **E**) Frequency of $FoxP3^+CD38^+$ T cells within the T_{regs} compartment ($CD4^+FoxP3^+$ T cells) was assessed in [(A) and (B)] tumor tissue and paired blood samples from patients bearing (A) breast cancer (Ca) and (B) muscle-invasive bladder cancer (MIBC); [(C) to (E)] nonlymphoid tissues, lymphoid tissues, and tumor tissue from B6 mice bearing 15 days subcutaneously established (C) B16-F10 melanoma, (D) YUMM1.7 melanoma, and (E) EL-4 thymoma. Adjacent bar plots represent cumulative data from 9 (A), 12 (B), 4 (C), 4 (D), and 4 (E) samples. (**F** to **H**) The fluorescence intensity of CD38 was assessed in T_{regs} from (F) B16-F10, (G) YUMM1.7, and (H) EL-4 thymoma bearing mice. The bar diagrams represent cumulative data from four [(F) to (H)] independent experiments. (**I**) *t*–Distributed stochastic neighbor embedding (*t*-SNE) representation highlights the five identified T_{regs} (*$CD3^+CD4^+FOXP3^+$*) subclusters derived from the single-cell RNA sequencing (scRNA-seq) clusters of *$CD3G^+$* T cells. (**J**) *t*-SNE plot showing single-cell transcription levels of key genes, with colors indicating their expression: blue for *CD3G* and *CD4*, green for *FOXP3*, and red for *CD38*. (**K**) Pie chart depicting the relative abundance of five identified T_{regs} clusters across the five breast cancer samples. (**L**) The heatmap displays the top 100 differentially expressed genes, ranked by log_2 fold change, across all identified T_{regs} clusters. *$P < 0.05$; ****$P < 0.0001$. FMO, fluorescence minus one.

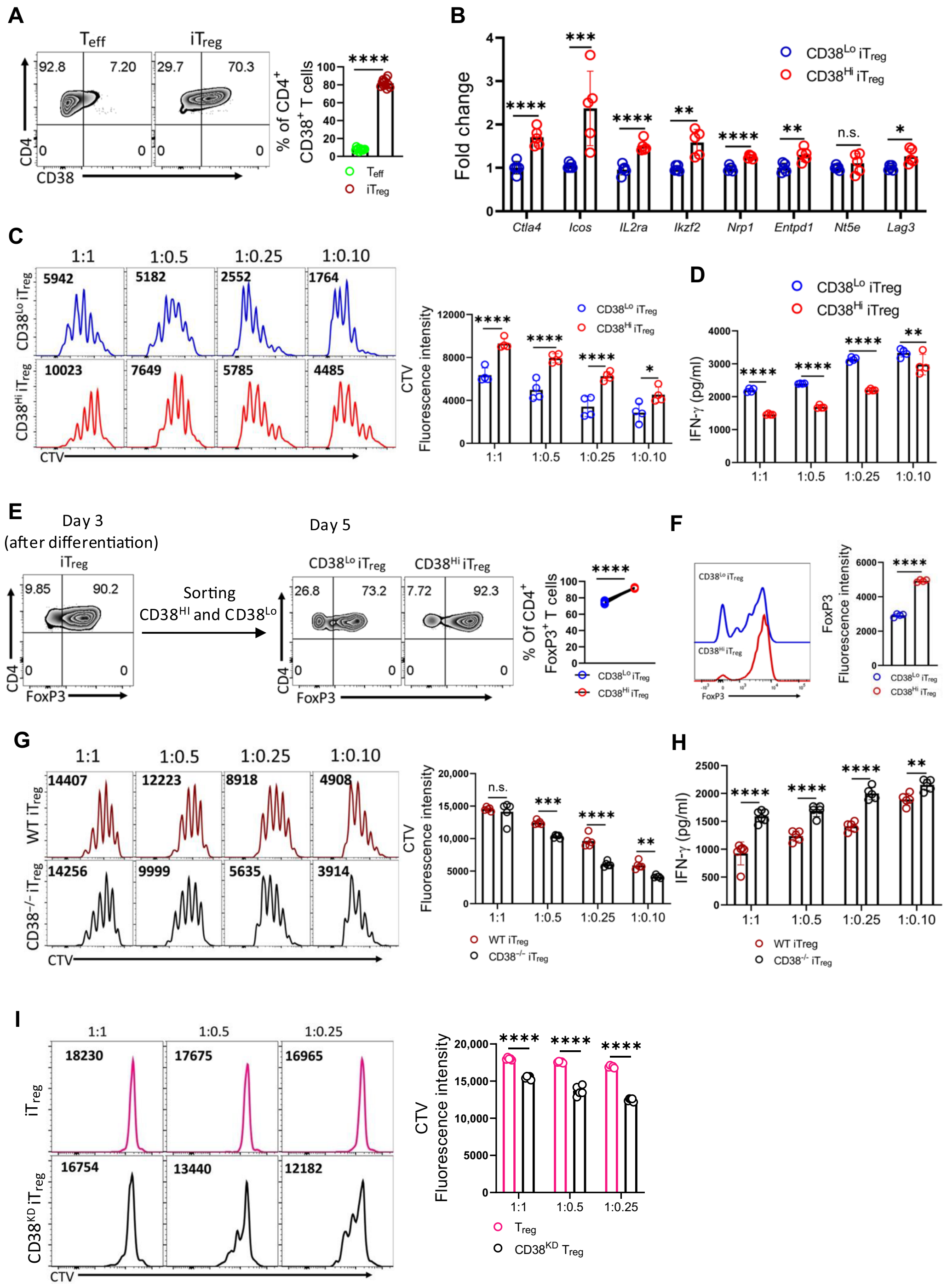

Fig. 2. CD38 endows T_{regs} with superior suppressive function. (**A**) Naïve $CD4^+$ T cells from B6 mice were differentiated into T_{eff} or T_{regs}, and CD38 expression was assessed. The bar diagram shows cumulative data from 11 experiments. (**B**) Quantitative polymerase chain reaction (qPCR) analysis of various genes in $CD38^{Hi}$ and $CD38^{Lo}$ iT_{regs}. Data are representative of five independent experiments. (**C**) CellTrace Violet (CTV)–labeled naïve T cells were activated with anti-CD3/CD28 (1 μg/ml each) in the presence of fluorescence-activated cell sorting (FACS)–sorted $CD38^{Hi}$ and $CD38^{Lo}$ iT_{regs} for 3 days. $CD8^+$ T cell proliferation was assessed via CTV dilution. Bar diagram shows data from four experiments. (**D**) Supernatant from (C) was used to measure interferon-γ (IFN-γ) levels. (**E**) $CD4^+FoxP3^+$ T cell frequency on day 3 of iT_{regs} differentiation (left) and after sorting into $CD38^{Hi}$ and $CD38^{Lo}$ subsets, followed by culturing in interleukin-2 (IL-2) for 2 days (right). The bar plot represents data from four experiments. (**F**) FoxP3 expression in $CD38^{Hi}$ and $CD38^{Lo}$ iT_{regs} after culturing in IL-2 for 2 days. The bar plot shows data from four independent experiments. (**G**) CTV-labeled naïve T cells were activated with anti-CD3/CD28 in the presence of wild-type (WT) and $CD38^{-/-}$ iT_{regs} for 3 days. $CD8^+$ T cell proliferation was assessed via CTV dilution. Adjacent bar diagram represents data from five independent experiments. n.s., not significant. (**H**) Supernatant from (G) was used to assess IFN-γ levels. (**I**) Human iT_{regs} transduced with either control shRNA or CD38 shRNA were cocultured with CTV-labeled naïve T cells in the presence of anti-CD3/CD28 for 3 days. $CD8^+$ T cell proliferation was assessed via CTV dilution. The adjacent bar diagrams represent cumulative data from five independent experiments. $*P < 0.05$; $**P < 0.01$; $***P < 0.005$; $****P < 0.0001$.

suppressing the proliferation and effector cytokine production of T cells (Fig. 2, G and H).

Last, we sought to determine whether iT_{regs} differentiated from human $CD4^+$ T cells also express CD38 and whether CD38 contributes to their suppressive function. $CD4^+$ T cells purified from the peripheral blood mononuclear cells (PBMCs) of healthy volunteers were differentiated into T_{effs} or iT_{regs} (fig. S2G), and CD38 expression was evaluated. Consistent with findings in murine iT_{regs}, CD38 expression was predominant on human iT_{regs} compared to that on T_{effs} (fig. S2H). Short hairpin RNA (shRNA)–mediated knockdown of CD38 in human iT_{regs} significantly reduced their suppressive function (Fig. 2I and fig. S2I), underscoring the critical role of CD38 in regulating human iT_{regs} functionality, mirroring the observations in murine iT_{regs}. Together, these findings suggest that CD38 expression on iT_{regs} is pivotal for their immunosuppressive potential and aids in maintaining FoxP3 expression, highlighting its critical role in T_{regs} stability and function.

$CD38^{Hi}$ iT_{regs} display restricted mitochondrial activity, essential for their suppressive function

Given the metabolic heterogeneity in iT_{regs} and its impact on their functional traits, we investigated whether $CD38^{Hi}$ and $CD38^{Lo}$ iT_{regs} are metabolically distinct. We found that glucose metabolism, assessed by measuring the extracellular acidification rate (ECAR), a measure of glycolysis, was significantly higher in $CD38^{Lo}$ iT_{regs} compared to that in $CD38^{Hi}$ iT_{regs} (Fig. 3, A to C). However, the expression of genes encoding various glycolytic enzymes was comparable between $CD38^{Lo}$ and $CD38^{Hi}$ iT_{regs}, except for *Ldha*, which showed significant up-regulation in $CD38^{Hi}$ iT_{regs} (fig. S3A). The data suggest that glycolytic enzyme levels were less likely to account for differential glucose metabolism in these cells, as reported recently in T_{regs} subsets having discrete proclivities for glucose metabolism (*8*). $CD38^{Hi}$ iT_{regs} are highly reminiscent of low-glucose–avid T_{regs} that have been reported to have superior suppressive function and FoxP3 stability (*8*).

Because oxidative metabolism was found to be critical in regulating T_{regs} function (*27*, *28*), we next determined the OXPHOS in $CD38^{Hi}$ and $CD38^{Lo}$ iT_{regs} by measuring their oxygen consumption rate (OCR). Contrary to earlier studies (*9*, *10*, *27*), we noted that $CD38^{Hi}$ iT_{regs}, despite having high suppressive potential, exhibited decreased mitochondrial respiration compared to $CD38^{Lo}$ iT_{regs}, indicating reduced mitochondrial activity in $CD38^{Hi}$ iT_{regs} (Fig. 3, D to G). Further analysis revealed that the observed reduction in mitochondrial OXPHOS in $CD38^{Hi}$ iT_{regs} was not due to the decreased expression of the electron transport chain (ETC) complexes (fig. S3B). Next, to confirm the role of CD38 in affecting mitochondrial metabolism in $CD38^{Hi}$ iT_{regs}, we measured OXPHOS in iT_{regs} differentiated from either WT or $CD38^{-/-}$ B6 mice. We observed that iT_{regs} differentiated from $CD38^{-/-}$ $CD4^+$ T cells had higher mitochondrial respiration compared to WT iT_{regs} (Fig. 3, H to K), suggesting that CD38 restricts mitochondrial activity in iT_{regs}. These observations highlight a complex metabolic regulation in iT_{regs} exerted by CD38 that limits both glycolysis and oxidative metabolism. Yet, the relevance of these metabolic features to the suppressive function of $CD38^{Hi}$ iT_{regs} was unclear.

Glycolysis and OXPHOS are coupled through the oxidation of pyruvate and its subsequent incorporation into the TCA cycle, generating reducing equivalents [reduced form of NAD^+ (NADH) and flavin adenine dinucleotide (FADH2)] for oxidation-reduction reactions in the ETC. Any impediment restricting the utilization of pyruvate in the TCA cycle and subsequently generating reducing equivalents for ETC could impinge upon the whole metabolic circuit. We argued that reduced metabolic activity in $CD38^{Hi}$ iT_{regs} could be due to their restricted mitochondrial activity, which might be important for their heightened suppressive capacity. To test this possibility, we took an indirect approach where different complexes of ETC were inhibited using pharmacological inhibitors, and the relevance of mitochondrial metabolism in regulating the suppressive capacity of T_{regs} was assessed. Inhibition of either complex I or complex III of the ETC using rotenone or antimycin A, respectively, at 48 hours of iT_{regs} differentiation, did not affect the viability or FoxP3 expression in iT_{regs} (fig. S3, C and D). Intriguingly, iT_{regs} differentiated in the presence of rotenone were highly immunosuppressive, as both the proliferation and interferon-γ (IFN-γ) production by T cells cocultured with rotenone-treated iT_{regs} were significantly reduced. Conversely, inhibition of complex III during iT_{regs} differentiation impaired their suppressive potential (Fig. 3, L and M). Complex III inhibition–mediated iT_{regs} dysfunction was contemplated, as the ETC was partially operative through complex I, leading to the accumulation of TCA cycle intermediates α-KG, succinate, and 2-hydroxyglutarate that promote T_{regs} dysfunction, as reported (*28*). To further confirm that the CD38-NAD^+ axis–mediated restriction of mitochondrial activity is crucial for the superior suppressive function of $CD38^{Hi}$ iT_{regs}, we repeated the experiments using WT and $CD38^{-/-}$ iT_{regs}. While $CD38^{-/-}$ iT_{regs} exhibited impaired suppressive potential, their suppressive function was significantly restored to levels comparable to that of WT iT_{regs} when differentiated in the presence of a complex I inhibitor (rotenone) but not a complex III inhibitor (antimycin A) (fig. S3E). These findings strongly suggest that the heightened suppressive function of $CD38^{Hi}$ iT_{regs} is driven by restricted mitochondrial activity regulated by the CD38-NAD^+ axis.

To elucidate whether rotenone-mediated hyper-immunosuppression was, in part, due to the reduced generation of TCA cycle intermediates, we performed metabolomics analysis. Although citrate levels were high, the downstream metabolites α-KG and succinate were significantly low in T_{regs} differentiated in the presence of rotenone (Fig. 3, N to R). Correspondingly, the OCR levels were markedly low in the rotenone-treated T_{regs}, while a modest increase in their ECAR levels was evident, likely to compensate for their bioenergetic needs (fig. S3, F and G).

Because mitochondrial impairment has been reported in $CD4^+$ T cells expressing CD38 (*20*), we investigated whether CD38-mediated mitochondrial dysfunction alone was sufficient to confer suppressive function on $CD4^+$ T cells, independent of FoxP3 expression. However, $CD4^+CD38^+FoxP3^-$ T cells did not exhibit any substantial suppressive function compared to iT_{regs} (fig. S3, H to J). Overall, these data indicate that mitochondrial oxidative metabolism is pivotal in regulating the suppressive function of T_{regs}, and CD38 limits oxidative metabolism, endowing T_{regs} with heightened immunosuppressive capacity.

CD38-mediated depletion of intracellular NAD^+ pool regulates the function and stability of T_{regs} through regulating mitochondrial metabolism

CD38 is a NADase and has been reported to maintain the intracellular NAD^+ pool (*29*). Intracellular NAD^+ content regulates diverse metabolic processes, including the TCA cycle, by acting as a coenzyme for various dehydrogenases, which catalyze the reduction of NAD^+ to NADH that is subsequently used in the oxidation-reduction

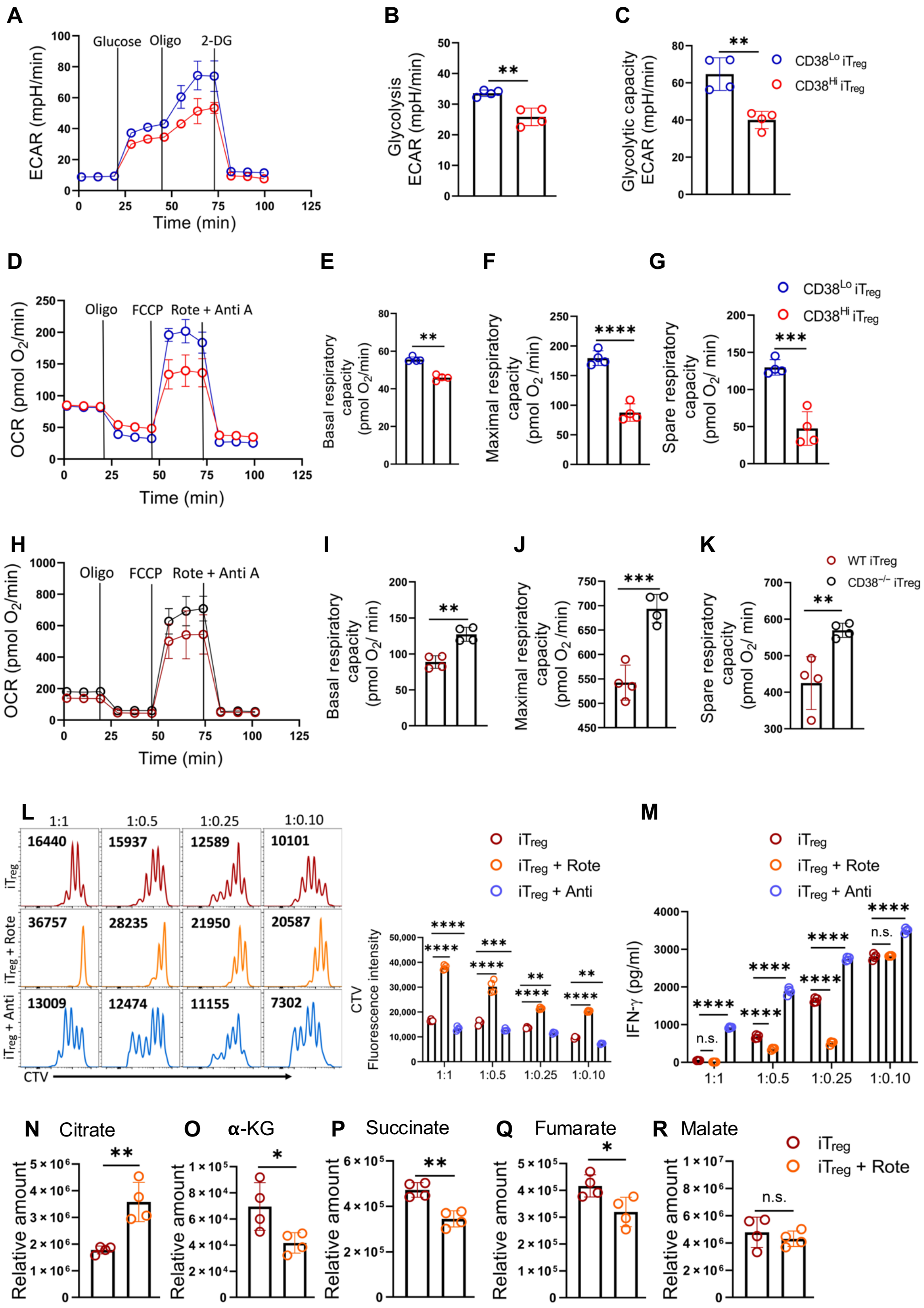

Fig. 3. CD38 expression restricts the mitochondrial metabolism in Treg. (**A** to **G**) iT_{regs} were sorted into $CD38^{Hi}$ and $CD38^{Lo}$ subsets and were used to determine: (A) extracellular acidification rate (ECAR) in response to glucose, oligomycin, and 2DG; (B) glycolysis rate after glucose addition; (C) glycolytic capacity; (D) oxygen consumption rate (OCR) under basal condition and in response to different inhibitors as indicated; (E) basal respiratory capacity; (F) maximal respiratory capacity; and (G) spare respiratory capacity. Data are representative of four [(A) to (G)] independent experiments. (**H** to **K**) WT and $CD38^{-/-}$ iT_{regs} were assessed for (H) OCR under basal condition and in response to the indicated inhibitors, (I) basal respiratory capacity, (J) maximal respiratory capacity, and (K) spare respiratory capacity. Data are representative of four [(H) to (K)] independent experiments. (**L** and **M**) iT_{regs} were differentiated in the presence or absence of rotenone (Rote; 1 μM, added at 48 hours) and antimycin A (Anti; 1 μM, added at 48 hours). After washing, they were cocultured with CTV-labeled T cells in the presence of a T cell activation cocktail (anti-CD3/CD28) for 3 days. (L) $CD8^+$ T cell proliferation was assessed via CTV dilution. The adjacent bar diagram shows data from four independent experiments. (M) Supernatant from (L) was used to measure IFN-γ levels. (**N** to **R**) Mass spectrometry (MS)–based determination of indicated intracellular metabolites in iT_{regs} differentiated in the presence or absence of rotenone (Rote; 1 μM, added at 48 hours). Data are representative of four independent experiments. $*P < 0.05$; $**P < 0.01$; $***P < 0.005$; $****P < 0.0001$.

reactions in the ETC (*30*). To elucidate whether CD38 affected mitochondrial metabolism by regulating the intracellular NAD^+ pool, we first checked NAD^+ content in $CD38^{Hi}$ and $CD38^{Lo}$ iT_{regs}. We found that NAD^+ levels were substantially low in $CD38^{Hi}$ iT_{regs}, albeit the comparable expression of genes involved in the biosynthesis of NAD^+ between $CD38^{Hi}$ and $CD38^{Lo}$ iT_{regs}, indicating that the reduced NAD^+ levels in $CD38^{Hi}$ iT_{regs} were a result of CD38-mediated utilization of NAD^+ (Fig. 4A and fig. S4A). Moreover, NAD^+ levels were also found to be low in T_{regs} compared to those in T_{effs} (fig. S4B). Next, we checked whether low NAD^+ content in $CD38^{Hi}$ iT_{regs} was commensurate with reduced mitochondrial metabolism. Metabolomics analysis revealed that the mitochondrial metabolites, including citrate, α-KG, fumarate, and malate levels, were significantly low $CD38^{Hi}$ in iT_{regs} compared to those in $CD38^{Lo}$ iT_{regs} (Fig. 4B).

To ascertain the role of NAD^+ in regulating mitochondrial activity and, subsequently, the suppressive function of iT_{regs} expressing high CD38, we replenished intracellular NAD^+ levels by supplementing NAD^+ precursor nicotinamide mononucleotide (NMN) during iT_{regs} differentiation. NMN supplementation did increase the intracellular NAD^+ levels in $CD38^{Hi}$ iT_{regs} but had no adverse effect on the viability or the differentiation of T_{regs} (fig. S4, C to E). NMN supplementation restored mitochondrial metabolism in $CD38^{Hi}$ iT_{regs}, as evidenced by the increased levels of various TCA cycle metabolites and improved mitochondrial respiration; however, it had no impact on the glycolysis of $CD38^{Hi}$ iT_{regs} (Fig. 4, C to G, and fig. S4F).

Next, we sought to determine whether restoring mitochondrial activity in $CD38^{Hi}$ iT_{regs} by replenishing their intracellular NAD^+ content would affect their suppressive function. NMN treatment markedly diminished the suppressive function of $CD38^{Hi}$ iT_{regs}, and the effect was found to be dose dependent (Fig. 4, H and I, and fig. S4, G and H). We found no reduction in cell viability or defect in T_{regs} differentiation even at a higher dose of NMN (fig. S4, I and J). Similar to NMN, the supplementation of nicotinamide riboside (NR), another precursor of NAD^+, also affected the suppressive potential of $CD38^{Hi}$ iT_{regs} (fig. S4, K and L). These data collectively indicate that CD38 expression in T_{regs} is essential for maintaining their intracellular NAD^+ levels, which, in turn, supports their mitochondrial metabolism. This process is likely crucial for the stability and functionality of T_{regs}.

Next, we aimed to elucidate the mechanism by which reduced mitochondrial activity facilitates the superior functionality and stability of $CD38^{Hi}$ iT_{regs}. Several studies have identified the demethylation of the *Foxp3* locus, particularly at three well-characterized conserved noncoding regions (CNS1 to CNS3), as critical epigenetic modifications that govern the differentiation, stability, and functionality of T_{regs} (*31–34*). Therefore, we first assessed the CpG methylation of the *Foxp3* locus. CNS2, along with CNS1 and CNS3, was hypomethylated in iT_{regs} compared to that in T_{effs} (fig. S4M). When comparing $CD38^{Hi}$ and $CD38^{Lo}$ iT_{regs}, we found that all three CNS regions were highly hypomethylated in $CD38^{Hi}$ iT_{regs} compared to those in $CD38^{Lo}$ iT_{regs}, underscoring the mechanistic basis for the superior stability and functionality of $CD38^{Hi}$ iT_{regs} (Fig. 4J).

Because the TCA cycle metabolite α-KG has been shown to regulate DNA methylation, including regions in the *Foxp3* gene locus (*11*), we sought to determine whether the maintenance of the hypomethylated state at the *Foxp3* gene locus in $CD38^{Hi}$ iT_{regs} was due to their reduced α-KG levels. To this end, we supplemented $CD38^{Hi}$ iT_{regs} cultures with cell-permeable α-KG and subsequently assessed methylation at the CNS regions and suppressive function. We observed that α-KG supplementation significantly increased methylation at CNS2 and CNS3 of the *Foxp3* locus and rendered $CD38^{Hi}$ iT_{regs} functionally impaired (Fig. 4, K and L). Notably, NMN-supplemented $CD38^{Hi}$ iT_{regs}, with increased levels of α-KG, also exhibited increased methylation at the CNS regions of the *Foxp3* locus (Fig. 4M). To further investigate whether hypermethylation induced by NMN or α-KG destabilizes FoxP3 expression, we assessed FoxP3 levels in $CD38^{Hi}$ iT_{regs} following supplementation. While FoxP3 expression remained unaffected at an early time point (24 hours post-treatment), a significant reduction was observed after an additional 2 days of culture, reinforcing the idea that NMN- or α-KG-mediated hypermethylation of the CNS regions predisposes $CD38^{Hi}$ iT_{regs} to FoxP3 loss and functional instability over time (fig. S4, N and O). Collectively, these data suggest that CD38 in iT_{regs} acts as a metabolic checkpoint, regulating intracellular NAD^+ content to ensure restricted mitochondrial metabolism and the generation of TCA cycle metabolites, particularly α-KG, which adversely affect T_{regs} stability and functionality.

$CD38^{Hi}$-NAD^{Lo} axis redirects lactate metabolism for the generation of PEP in T_{regs}

Uptake and metabolism of lactate is a hallmark of intratumoral T_{regs} (*7*, *8*). Given that CD38 expression plays a distinctive role in rewiring the metabolic traits of iT_{regs}, we explored whether this axis contributes to the efficient metabolism of lactate, enabling iT_{regs} to retain their identity and functionality. Consistent with the previous study (*8*), we observed that iT_{regs} had a greater propensity to uptake lactate compared to T_{effs} (fig. S5A). The lactate uptake was comparable between $CD38^{Hi}$ and $CD38^{Lo}$ iT_{regs} (fig. S5B). Because lactate metabolism has been shown to profoundly influence T_{reg} functionality, we hypothesized that despite comparable lactate uptake, $CD38^{Hi}$ and $CD38^{Lo}$ iT_{regs} might be differentially equipped to metabolize lactate, leading to distinct functional outcomes.

The metabolic fate of lactate is usually determined by two enzymes: pyruvate dehydrogenase (PDH) and PC (*15*). PDH converts lactate-derived pyruvate to acetyl coenzyme A (CoA) for its further incorporation into the TCA cycle, whereas PC promotes the conversion of lactate-derived pyruvate to OAA, which is subsequently converted to PEP by PEPCK to be used in gluconeogenesis (*15*). To assess the contribution of these enzymes in lactate metabolism in $CD38^{Hi}$ and $CD38^{Lo}$ iT_{regs}, we first traced the metabolic fate of lactate by incubating the cells with uniformly labeled $^{13}C_3$ L-lactate for 18 hours in glucose-free medium. We tracked ^{13}C incorporation into acetyl CoA and PEP, reflecting lactate's commitment to entering the TCA cycle or gluconeogenesis, respectively. Notably, $CD38^{Hi}$ iT_{regs} exhibited significantly higher allocation of ^{13}C to PEP (M+3), while $CD38^{Lo}$ iT_{regs} showed an increase, albeit nonsignificant, in ^{13}C labeling in acetyl-CoA (M+2) (Fig. 5, A and B). These findings suggest a differential metabolic routing of lactate, with $CD38^{Hi}$ iT_{regs} favoring gluconeogenesis, a feature also observed in intratumoral T_{regs} (*8*). In contrast, $CD38^{Lo}$ iT_{regs} appear to preferentially engage the TCA cycle. Additionally, consistent with previous reports linking low NAD^+ levels to reduced lactate oxidation into pyruvate (*7*), we observed a modest, although nonsignificant, decrease in ^{13}C incorporation into pyruvate in $CD38^{Hi}$ iT_{regs} compared to that in $CD38^{Lo}$ iT_{regs} (Fig. 5C).

Next, to assess whether PC-mediated metabolic routing of lactate in gluconeogenesis was key for maintaining the functionality of

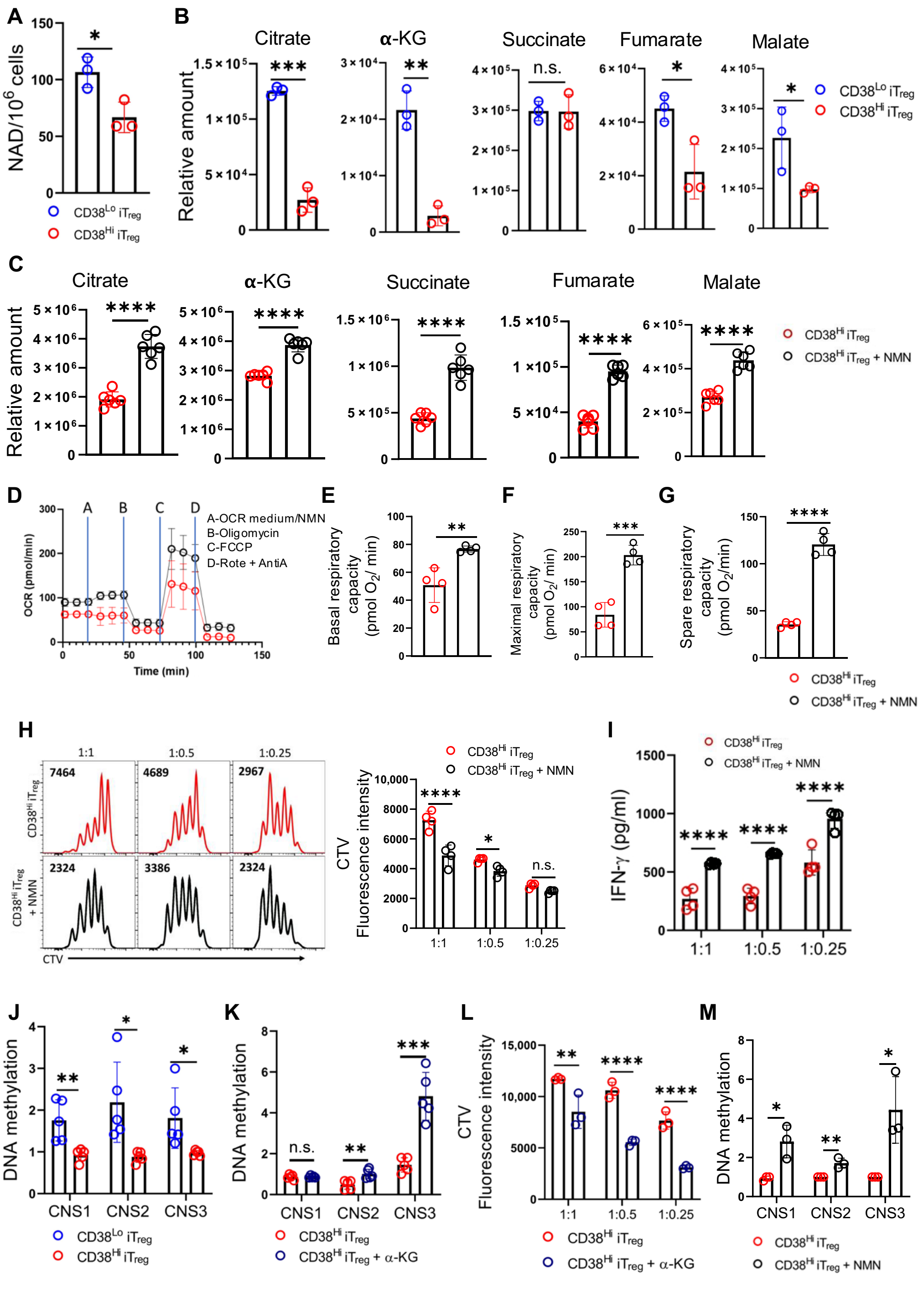

Fig. 4. CD38-NAD$^+$ axis regulates the mitochondrial metabolism and suppressive function in T$_{regs}$. (**A** and **B**) iT$_{regs}$ sorted into CD38^{Hi} and CD38^{Lo} subsets were used to determine: (A) intracellular NAD$^+$ levels and (B) indicated intracellular metabolites using MS. (**C** to **G**) FACS-sorted CD38^{Hi} iT$_{regs}$ were cultured overnight in the presence or absence of NMN (500 μM) and were used for (C) determining the indicated intracellular metabolites using MS, (D) OCR under basal condition and in response to the indicated inhibitors, (E) basal respiratory capacity, (F) maximal respiratory capacity, and (G) spare respiratory capacity. (**H** to **I**) CD38^{Hi} iT$_{regs}$ cultured overnight in the presence or absence of NMN were cocultured with CTV-labeled naïve T cells in the presence of a T cell activation cocktail (anti-CD3/CD28) for 3 days and assessed for (H) responder CD8$^+$ T cell proliferation via CTV dilution and (I) measuring IFN-γ levels from the supernatant of the experiment shown in (H). (**J**) qPCR-based analysis of CpG methylation at CNS regions of the *Foxp3* locus was performed on CD38^{Hi} and CD38^{Lo} iT$_{regs}$. (**K** and **L**) CD38^{Hi} iT$_{regs}$ cultured overnight in the presence or absence of cell-permeable α-KG (100 μM) were used to assess (K) CpG methylation at CNS regions of the *Foxp3* locus and (L) suppressive potential by measuring responder CD8$^+$ T cell proliferation after 3 days of coculture with naïve T cells under T cell activation conditions. (**M**) qPCR-based analysis of CpG methylation at CNS regions of the *Foxp3* locus was performed in CD38^{Hi} iT$_{regs}$ supplemented with NMN. The data are representative of three (A), three (B), six (C), four [(D) to (I)], five [(J) and (K)], and three [(L) and (M)] independent experiments. *$P < 0.05$; **$P < 0.01$; ***$P < 0.005$; ****$P < 0.0001$.

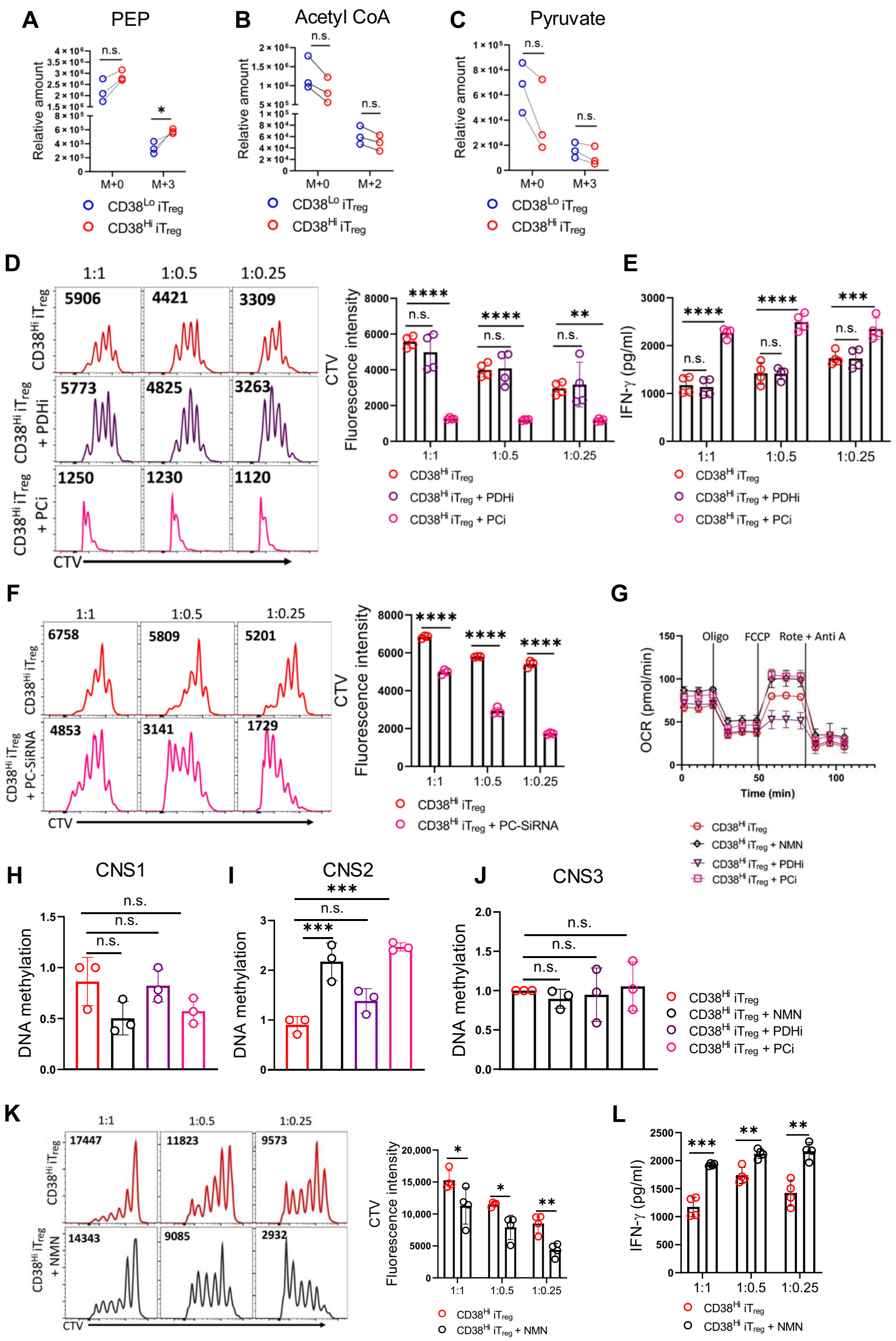

Fig. 5. CD38 is crucial for the metabolic adaptability of T_{regs} to use lactate and maintain suppressive function. (**A** to **C**) Isotopologue distribution for (A) PEP, (B) acetyl CoA, and (C) pyruvate in $CD38^{Hi}$ and $CD38^{Lo}$ iT_{regs} overnight cultured in glucose-free medium containing $^{13}C_3$ L-lactate. (**D** and **E**) iT_{regs} were differentiated in glucose-free, L-lactate–containing medium and sorted into the $CD38^{Hi}$ population. These were then treated overnight with the indicated inhibitors in the same L-lactate–containing medium and were used for coculturing with CTV-labeled naïve T cells in the presence of a T cell activation cocktail (anti-CD3/CD28) for 3 days and assessed for (D) $CD8^+$ T cell proliferation via CTV dilution and (E) measuring IFN-γ levels from the supernatant of the experiment shown in (D). (**F**) T cell proliferation was assessed via CTV dilution after 3 days of coculturing naïve T cells with $CD38^{Hi}$ iT_{regs} that were differentiated in glucose-free, L-lactate–containing medium, following the indicated RNA interference–mediated knockdown. (**G** to **J**) iT_{regs} were differentiated in glucose-free, L-lactate–containing medium and sorted into the $CD38^{Hi}$ population before being treated overnight with NMN and indicated inhibitors. Cells were then assessed for (G) OCR under basal condition and in response to the indicated inhibitors, (H) CpG methylation at CNS1, (I) CpG methylation at CNS2, and (J) CpG methylation at CNS3 regions of the *Foxp3* locus. (**K** and **L**) NMN-treated and untreated $CD38^{Hi}$ iT_{regs} differentiated in glucose-free, L-lactate–containing medium were cocultured with CTV-labeled naïve T cells for 3 days in the presence of anti-CD3/CD28. (K) Proliferation of $CD8^+$ T cells was assessed by CTV dilution, and (L) supernatant from (K) was used to measure IFN-γ levels. The data are representative of three [(A) to (C)], three [(F) to (J)], four [(D) and (E)], and four [(K) and (L)] independent experiments. $^*P < 0.05$; $^{**}P < 0.01$; $^{***}P < 0.005$; $^{****}P < 0.0001$.

$CD38^{Hi}$ iT_{regs}, we blocked the enzyme and checked the functionality. Notably, inhibition of PC in $CD38^{Hi}$ iT_{regs} cultured in lactate medium or even in complete medium (CM) significantly curbed their function, while no such impairment in the functionality was evident upon PDH inhibitor treatment (Fig. 5, D and E, and fig. S5, C and D). The pivotal role of gluconeogenesis in contributing to the functionality of $CD38^{Hi}$ iT_{regs} was further confirmed by using RNA interference targeting PC and PEPCK (Fig. 5F and fig. S5E).

To explore the mechanism underpinning the routing of lactate into gluconeogenesis by PC in $CD38^{Hi}$ iT_{regs}, we examined whether the intracellular NAD^+ pool plays a decisive role. Because PDH is an NAD^+-dependent enzyme, we postulated that CD38, through depleting the NAD^+ pool, enforces lactate utilization through PC rather than PDH. To delineate this, we supplemented NMN while culturing $CD38^{Hi}$ iT_{regs} in lactate medium and checked their functionality. We observed that NMN supplementation in $CD38^{Hi}$ iT_{regs} cultured in lactate-rich medium improved their mitochondrial OXPHOS and reversed DNA hypomethylation at the *Foxp3* locus (Fig. 5, G to J). A similar effect was seen when PC, but not PDH, was inhibited in $CD38^{Hi}$ iT_{regs} cultured in lactate medium (Fig. 5, G to J). Moreover, commensurate with increased OXPHOS and CNS methylation, NMN supplementation alleviated the suppressive function of $CD38^{Hi}$ iT_{regs} (Fig. 5, K and L). Last, we assessed OXPHOS in $CD38^{-/-}$ iT_{regs}, which have intrinsically elevated intracellular NAD^+ levels, under lactate-rich conditions. Our findings revealed that CD38 deficiency enhanced OXPHOS in the presence of lactate (fig. S5, F to I), highlighting the critical role of the CD38-NAD^+ axis in regulating lactate metabolism in T_{regs}. Together, these findings establish the CD38-NAD^+ axis as a crucial determinant of T_{reg} metabolic adaptability, enabling them to thrive in the lactate-rich TME.

Small-molecule targeting of CD38 alleviates T_{regs} function and improves the antitumor response in mice

After identifying CD38 as essential for maintaining iT_{regs} function and stability in a lactate-rich environment, we next sought to extend our findings to the TME. To this end, gp100-reactive T cell receptor (TCR) transgenic $CD8^+$ T cells (Pmel T cells) were activated with their cognate antigen for 3 days and adoptively cotransferred (at a 1:1 ratio) with either WT or $CD38^{-/-}$ iT_{regs}, which were CellTrace Violet (CTV)–labeled, into B16-F10 melanoma-bearing mice with tumors established subcutaneously for 10 days. Upon retrieval on 48 hours after adoptive transfer, Pmel T cells, when transferred without iT_{regs}, were notably abundant at the tumor site, more so than in the spleen and DLN, likely due to antigen-driven expansion. Cotransferring these cells with WT iT_{regs} significantly reduced their frequency in the TME. However, when Pmel T cells were cotransferred with $CD38^{-/-}$ iT_{regs}, only a modest reduction was observed (Fig. 6A). Consistent with these findings, analysis of the transferred T_{regs} population revealed a substantial reduction in the frequency of $CD38^{-/-}$ iT_{regs} at the tumor site compared to that of WT iT_{regs}, while their frequencies in the spleen and DLN remained comparable (Fig. 6B). These findings indicate that CD38 deficiency impairs T_{regs} function in the TME, thereby permitting greater proliferation of Pmel T cells.

Given the critical role of CD38 in T_{regs} function and stability, we next explored whether small-molecule inhibition of CD38 could enhance the antitumor response. To investigate this, we first tested this approach on iT_{regs} differentiated in vitro in the presence of tumor-derived supernatant (TDS). We observed that iT_{regs} differentiated with TDS were modestly but significantly more suppressive than those differentiated in CM. Inhibition of CD38 with 78c (referred to as CD38i hereafter) reduced the suppressive function of iT_{regs} differentiated in both TDS and CM (Fig. 6C). The functional impairment was more pronounced in iT_{regs} differentiated in TDS compared to that in CM, suggesting that T_{regs} undergo distinct metabolic adaptations depending on carbon sources, which renders them differentially susceptible to CD38 inhibition–mediated functional impairment. Consistent with these findings, $CD38^{-/-}$ iT_{regs} differentiated in 30% TDS exhibited profound functional impairment compared to their counterparts differentiated in CM (fig. S6A).

Next, we assessed the methylation status of the *Foxp3* locus in CD38i-treated iT_{regs}. CD38i treatment induced hypermethylation in the CNS2 region of the *Foxp3* locus in iT_{regs} differentiated under both CM and 30% TDS conditions. The hypermethylation of CNS2 was more pronounced in iT_{regs} differentiated in 30% TDS, further supporting the observed severe functional impairment in these cells following CD38 inhibition (Fig. 6D).

Last, considering the established role of hypoxia in promoting T_{regs} functionality within the TME (*35*, *36*), we evaluated the effect of CD38 inhibition on iT_{regs} differentiated under hypoxic conditions. Similar to our observations with TDS-cultured iT_{regs}, CD38 inhibition significantly impaired the suppressive function of iT_{regs} differentiated in hypoxic conditions (fig. S6B).

To evaluate the therapeutic efficacy of CD38i, we subcutaneously engrafted the YUMM1.7 mouse melanoma tumor, which carries human-relevant mutations (BrafV600E; $Cdkn2a^{-/-}$; $Pten^{-/-}$; Tyrosinase:CreERT2), in B6 mice. We treated the mice with CD38i once a week until the tumor volume in vehicle control–treated mice reached the humane endpoint. We observed that CD38i treatment significantly delayed tumor growth (Fig. 6E). Analysis of the T_{regs} population revealed that CD38i treatment substantially reduced the frequency of intratumoral T_{regs} without affecting T_{regs} frequencies in the spleen or DLN (Fig. 6F). The reduction of intratumoral T_{regs} improved the $CD8^+$ T_{eff}-to-T_{regs} ratio at the tumor site (Fig. 6G) and substantially augmented the prevalence of multifunctional $CD8^+$ T cells at the tumor site (Fig. 6, H to J).

To further investigate whether CD38i directly affected the stability of intratumoral T_{regs}, we repeated the experiment in another cohort of YUMM1.7 tumor-bearing mice. The day before euthanasia, we administered 5-bromo-2′-deoxyuridine (BrdU) intraperitoneally. BrdU incorporation analysis revealed that $CD38^{Hi}$ T_{regs} in the TME were more proliferative than $CD38^{Lo}$ T_{regs} (Fig. 6K). CD38i treatment markedly diminished their proliferation (Fig. 6L). These data suggest that CD38 inhibition renders intratumoral T_{regs} metabolically unfit to thrive in the TME, leading to a loss of stability and reduced proliferation.

DISCUSSION

Understanding how immune cells metabolically adapt to their local microenvironment, which influences their function and persistence, is becoming crucial for devising strategies to modulate the host immune response to various pathological conditions. Previous studies have demonstrated the metabolic plasticity of T_{regs}, enabling them to reorient their metabolic traits in a tissue-specific manner (*7*, *13*, *37*). Notably, intratumoral T_{regs} have shown metabolic reliance to use lactic acid, thereby retaining their cellular identity and functionality (*7*, *8*). However, the precise mechanisms that enable intratumoral T_{regs} to rewire their metabolism to use lactic acid, supporting their

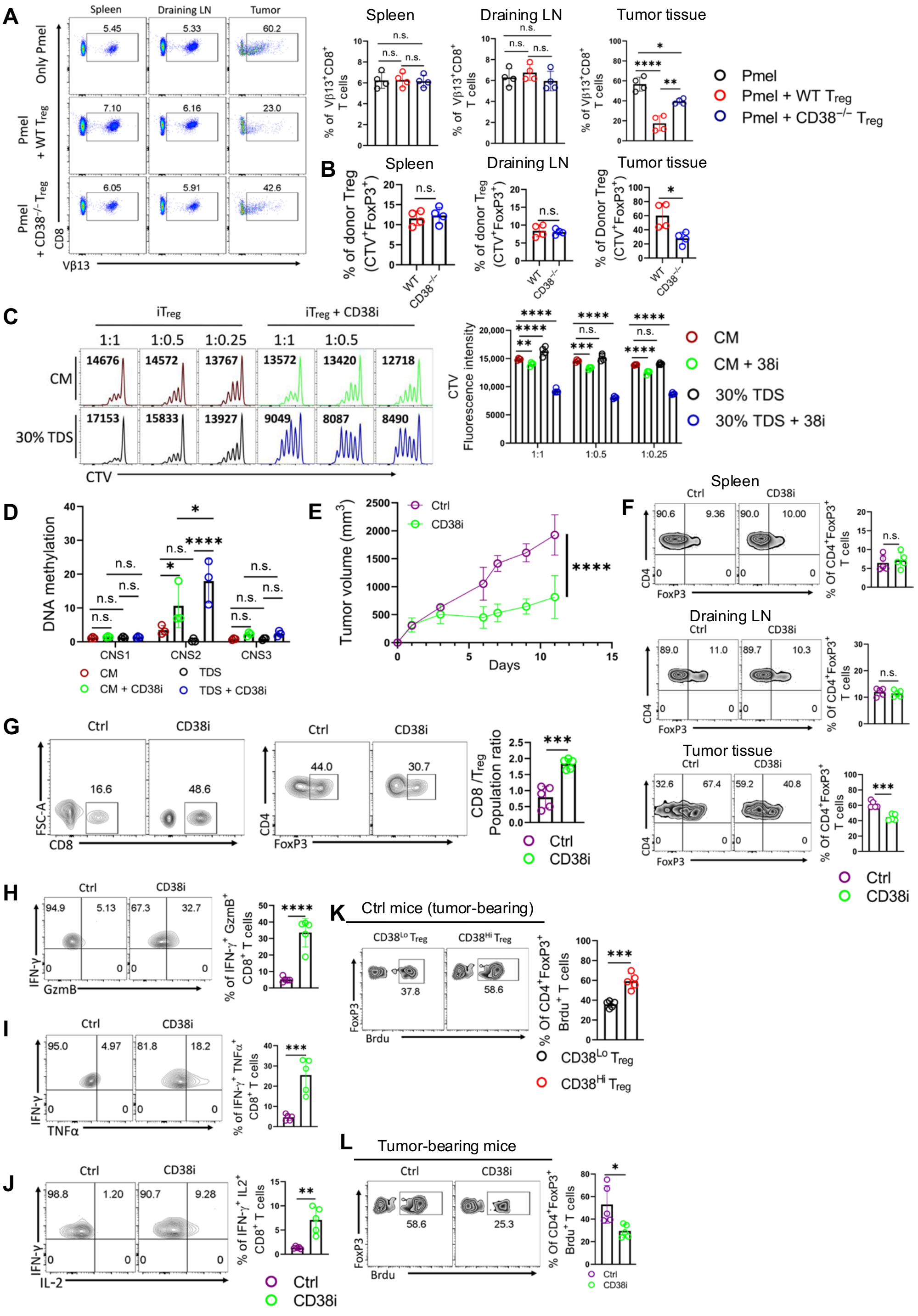

Fig. 6. Small-molecule targeting of CD38 impairs intratumoral T_{regs} and elicits antitumor response. (**A**) Frequency of adoptively transferred Pmel ($CD8^+v\beta13^+$) T cells from mice bearing 10 days subcutaneously established B16F10 melanoma. The adjacent bar represents data from $n = 4$ mice. (**B**) The bar diagrams depict the frequency of donor T_{regs} population ($Foxp3^+$ CTV^+) from the tumor-bearing mice shown in (A). (**C** and **D**) iT_{regs} were differentiated either in complete medium (CM) or in 30% TDS in the presence or absence of CD38i and were assessed for (C) evaluating suppressive potential by checking the proliferation of CTV-labeled $CD8^+$ T cells from the coculture and (D) CpG methylation at CNS1, CNS2, and CNS3 regions of the *Foxp3* locus. The data are representative of four (C) and three (D) independent experiments. (**E** to **J**) C57BL/6 mice ($n = 5$ mice per group) with subcutaneously established YUMM1.7 melanoma tumor were treated either with vehicle control or CD38i (15 mg/kg, intraperitoneal, once a week) and evaluated for (E) tumor growth; (F) T_{regs} frequency at the spleen, DLN, and tumor site; (G) assessment of T_{eff} ($CD8^+$ T cells) to T_{regs} ratio at the tumor site; and [(H) to (J)] the ability of $CD8^+$ T cells from the tumor site to coproduce different effector cytokines upon restimulation in vitro. TNF-α, tumor necrosis factor–α. (**K** and **L**) BrdU incorporation in (K) $CD38^{Lo}$ and $CD38^{Hi}$ intratumoral T_{regs} and (L) intratumoral T_{regs} from Ctrl and CD38i-treated mice. The representative data are from five [(E) to (L)] independent samples. *$P < 0.05$; **$P < 0.01$; ***$P < 0.005$; ****$P < 0.0001$. FSC-A, forward scatter area.

functionality in the TME, remain unclear. The present study reveals that the CD38-NAD$^+$ axis in T_{regs} plays a pivotal role in directing the metabolic routing of lactate, which appears critical for ensuring stable function and survival of T_{regs} in the TME.

While T_{regs} exhibit tissue-specific metabolic adaptations, the process within the TME appears to be more complex, largely due to the unique metabolic characteristics observed in intratumoral T_{regs} (*7*, *9*, *10*). Emerging evidence suggests that lactate serves as the primary carbon source for intratumoral T_{regs} (*8*). Therefore, to effectively metabolize lactate and support their function and survival in the TME, these cells must have adopted mechanisms distinct from those of peripheral T_{regs}, which usually rely on carbon sources other than lactate (*27*). A recent study investigating the metabolic flux of sodium lactate in intratumoral T_{regs} uncovered an intriguing observation: Intratumoral T_{regs} tend to generate PEP from TCA cycle–derived malate and fuel gluconeogenesis (*8*). This finding suggests that the steady incorporation of lactate-derived pyruvate in the mitochondrial TCA cycle may be constrained in intratumoral T_{regs}, as a fraction of malate is transported out of mitochondria to fuel gluconeogenesis. Congruently, studies from other groups have also shown compromised mitochondrial function and fitness in intratumoral T_{regs}, with mitochondrial aberrations found to enhance their suppressive function (*9*, *11*). Our data align with the previous studies by placing CD38 as a critical regulator of mitochondrial activity in intratumoral T_{regs}. We found that the restricted mitochondrial activity in T_{regs} was, in part, due to the CD38-mediated depletion of the intracellular NAD$^+$ content. We posited that the reduced NAD$^+$ content in CD38-expressing T_{regs} impeded their ability to efficiently drive the TCA cycle, as the essential dehydrogenase reactions were constrained by the limited supply of the critical coenzyme, NAD$^+$. We observed that T_{regs} with high CD38 expression exhibited reduced levels of various TCA cycle metabolites, which could be ameliorated by replenishing the intracellular NAD$^+$ levels by supplementing NAD$^+$ precursors NMN and NR. CD38 has long been considered an ectonucleotidase due to its type II orientation, where its catalytic domains face extracellularly. However, recent findings suggest a type III orientation, with the catalytic domain facing the cytoplasm (*38*, *39*). This dual orientation provides a mechanism for CD38's regulation of intracellular NAD$^+$ levels, as observed in T_{regs} and other cell types (*40*). In the present study, we observed that CD38-mediated regulation of intracellular NAD$^+$ levels affects the suppressive function of T_{regs} by modulating TCA cycle–derived α-KG production, which, in turn, affects iT_{regs} stability by influencing methylation in the CNS regions of the *Foxp3* locus. The notion was ascertained by assessing the stability of iT_{regs} differentiated in the presence of NMN and α-KG, which rendered iT_{regs} more prone to losing FoxP3 expression over extended culture periods.

Our data shed light on a very crucial aspect of T_{regs} metabolism in tumor. We found that restricted mitochondrial metabolism was critical for T_{regs} to keep the TCA cycle generated α-KG levels in check, which has been reported to affect the stability and functionality of T_{regs} by promoting methylation at the *Foxp3* locus (*11*). When T_{regs} were cultured in lactate medium or tumor-conditioned media (TCM) to mimic the carbon source available in TME, CD38 primarily directed the metabolic routing of lactate-derived pyruvate toward PEP rather than their complete oxidation in the TCA cycle, due to maintaining low NAD$^+$ levels that affect the TCA cycle. Moreover, we noted that PC-mediated conversion of pyruvate to OAA was more favored in the T_{regs} cultured in lactate, indicating that CD38-mediated low NAD$^+$ content rewires the metabolic features of T_{regs} to undergo alternative metabolic pathway supporting gluconeogenesis. This metabolic diversion appeared extremely critical for T_{regs} fed on lactate, as increasing the intracellular NAD$^+$ contents reoriented the lactate-derived pyruvate entry into the TCA cycle, leading to markedly enhanced methylation at the *FoxP3* locus and subsequently affecting the functionality of T_{regs}. It is important to note that, NAD$^+$ replenishment–mediated metabolic modulation, leading to the functional impairment of CD38-expressing T_{regs}, was highly apparent in T_{regs} cultured in the lactate or TCM compared to that in T_{regs} cultured in the CM. These discrepancies could be due to the discrete metabolic features adopted by T_{regs} based on the nutrients available in the milieu, as reported earlier (*8*, *41*, *42*).

Last, the study investigated the therapeutic potential of targeting CD38 to enhance antitumor immunity. Pharmacological inhibition of CD38 in tumor-bearing mice resulted in a significant reduction of intratumoral T_{regs}, an improved T_{eff}-to-T_{regs} ratio, and delayed tumor growth. These findings suggest that CD38 inhibition could be a viable strategy to disrupt T_{regs}-mediated immunosuppression in the TME by affecting their metabolic adaptability. Notably, CD38 inhibition selectively disrupted the proliferation of CD38-expressing intratumoral T_{regs} while leaving peripheral T_{regs} unaffected. Similar to our observations, targeting CD38$^+$ T_{regs} with the CD38 monoclonal antibody isatuximab in patients with multiple myeloma has been shown to alleviate T_{regs}-mediated suppression and concurrently improve the effector response of natural killer (NK) and CD8$^+$ T cells, without any systemic toxicity (*22*). Beyond T_{regs}, CD38 has also been implicated in regulating the function of myeloid-derived suppressor cells and terminally exhausted T cells, both of which are critical in dampening the durable antitumor response (*17*, *43*). Therefore, we propose that small-molecule targeting of CD38 could mitigate the immunosuppressive milieu at the tumor site, fostering a favorable environment for T_{effs} and NK cells to mount an effective antitumor response.

Overall, the results underscore the critical role of CD38 in modulating the function and metabolic adaptation of T_{regs} within tumors. By regulating NAD$^+$ levels and mitochondrial activity, CD38 ensures the stability and suppressive capacity of T_{regs}, which, in turn, supports tumor growth. The study's findings pave the way for potential therapeutic interventions targeting CD38 to modulate T_{regs} function and improve antitumor immunity.

MATERIALS AND METHODS

Mice

C57BL/6 mice were bred and housed in a pathogen-free facility at the CSIR–Indian Institute of Chemical Biology (IICB). CD38$^{-/-}$ mice were obtained from the Jackson Laboratory (Bar Harbor, MA). All experimental protocols were approved by the Institutional Animal Ethics Committee, CSIR-IICB, India (ref no. IICB/AEC/Meeting/Sep/2019/13). Both male and female mice, aged 6 to 8 weeks, were randomly selected for experimentation. In tumor control experiments, an equal number of male and female mice of the same age group were included.

Human samples

Surgically resected tumor tissue (1 to 2 g in normal saline) and peripheral blood (3 to 4 ml in heparin tube) from chemo naïve patients

with MIBC and breast cancer [both triple-negative breast cancer (TNBC) and non-TNBC] were collected by medical personnel on obtaining written informed consent from all human patients, with ethical approval from the Institutional Review Board (IRB) of IPGME&R and SSKM Hospitals, Kolkata India (ref no. IPGME&R/IEC/2020/624) and R.G. Kar Medical College and Hospital, Kolkata, India (memo no. RKC/892 dated 15 June 2023), respectively. The use of human samples complied with the safety regulations of CSIR-IICB, Kolkata, India.

Cell lines

B16-F10 and YUMM1.7 melanoma cell lines were obtained from American Type Culture Collection. EL4 was obtained from the National Center for Cell Science, Pune, India. All cell lines were confirmed to be free of mycoplasma contamination using the MycoAlert Mycoplasma Detection Kit (Lonza) before use. Cells between the four to six passages were used for all the in vivo experiments.

Preparation of TDS

YUMM1.7 melanoma cells (0.75×10^6 per mouse) were injected subcutaneously into the flanks of C57BL/6 WT mice. Mice were euthanized once the tumor volume reached 2000 mm^3. Tumors were dissociated into a single-cell suspension and plated at a concentration of 1×10^7 cells/ml in complete RPMI 1640 medium. After overnight incubation in a CO_2 incubator, the culture medium was centrifuged, filtered, and stored at −80°C for future use.

T_{regs} differentiation and culture conditions

Naïve CD4$^+$ T cells were isolated from splenocytes of 6- to 8-week-old mice using the Dynabeads Untouched Mouse CD4$^+$ T Cell Isolation Kit (Thermo Fisher Scientific, USA). These cells were differentiated into iT_{regs} [using TGF-β (5 ng/ml), interleukin-2 (IL-2; 300 U/ml), anti-mouse IFN-γ (10 μg/ml), and anti-mouse IL-4 (10 μg/ml)] or T_{effs} [with IL-2 (100 U/ml) and IL-12 (10 ng/ml)] in the presence of plate-bound mouse anti-CD3 (5 μg/ml) and anti-CD28 (5 μg/ml). Differentiation occurred in complete RPMI 1640 medium supplemented with 10% fetal bovine serum (FBS; Thermo Fisher Scientific, USA), 1% penicillin-streptomycin (HiMedia Laboratories, India), and 55 μM β-mercaptoethanol (β-ME) (Thermo Fisher Scientific, USA), under 5% CO_2 at 37°C in a humidified incubator for 3 days. In some experiments, on day 2 of iT_{reg} differentiation, cells were treated with specific inhibitors (1 μM rotenone and 1 μM antimycin) and harvested on day 3 for downstream assays. In other experiments, iT_{regs} were fluorescence-activated cell sorting (FACS)–sorted on day 3 into CD38^{Hi} and CD38^{Lo} populations and maintained in IL-2 (100 U/ml) until use. In some cases, FACS-sorted CD38^{Hi} iT_{regs} were treated overnight with NMN (500 μM), NR (400 μM), or dimethyl α-KG (100 μM), then thoroughly washed with phosphate-buffered saline (PBS), and used in subsequent assays. To assess Foxp3 stability, CD38^{Hi} iT_{regs} were treated overnight with either NMN (500 μM) or dimethyl α-KG (100 μM) and then cultured for an additional 2 days in the presence of IL-2 (100 U/ml).

For lactate-fed iT_{regs} studies, cells were differentiated in either CM or glucose-free medium supplemented with 10 mM L-lactate. On day 3, sorted CD38^{Hi} iT_{regs} were cultured overnight in their respective medium with IL-2 (100 U/ml), with or without inhibitors (100 μM PCi or 100 μM PDHi). In some cases, 3-day activated iT_{regs} were further cultured for two additional days in either 30% TDS-containing medium or CM, along with CD38i (5 μM), NMN (500 μM), or vehicle control.

For some studies, naïve CD4$^+$ T cells were differentiated into iT_{regs} under hypoxic (1% O_2) or normoxic (20% O_2) conditions for 3 days and subsequently used in assays.

In other experiments, purified naïve CD4$^+$ T cells were activated using plate-bound anti-CD3 (5 μg/ml) and anti-CD28 (2 μg/ml) in the presence of IL-2 (100 U/ml) in complete RPMI 1640 medium supplemented with 10% FBS for 72 hours. After activation, sustained TCR stimulation was provided using plate-bound anti-CD3 (5 μg/ml) for up to 7 days. Cells were passaged every 48 hours and maintained in the presence of IL-2 (25 U/ml). Chronically expanded T cells were used in subsequent assays.

For human T_{eff} or iT_{regs} differentiation, PBMCs were isolated from buffy coats of healthy human donors using Ficoll-Hypaque gradient centrifugation. The buffy coats were anonymized before use, and all samples were handled in accordance with IRB approval (no. IICB/IRB/2020/2P). Purified naïve CD4$^+$ T cells were differentiated into iT_{regs} [in the presence of TGF-β (5 ng/ml), IL-2 (300 U/ml), anti-human IFN-γ (10 μg/ml), and anti-human IL-4 (10 μg/ml)] or T_{effs} [in the presence of IL-2 (100 U/ml)]. Differentiation was performed with plate-bound human anti-CD3 (5 μg/ml) and anti-CD28 (5 μg/ml) in complete RPMI 1640 medium supplemented with 10% FBS (Thermo Fisher Scientific, USA) and 1% penicillin-streptomycin (HiMedia Laboratories, India) under 5% CO_2 at 37°C in a humidified incubator for 3 days. In all experiments, iT_{regs} were thoroughly washed with PBS before being used for coculture.

T cell suppression assay

Naïve T cells were isolated from the spleens of WT C57BL/6 mice and labeled with CTV using the CellTrace Violet Cell Proliferation Kit (Thermo Fisher Scientific, USA) following the manufacturer's instructions. The CTV-labeled T cells were then plated at a density of 0.2×10^6 cells per well in a 96-well plate, along with iT_{regs} at varying ratios: 0.2×10^6 cells per well for a 1:1 ratio, 0.1×10^6 cells per well for 1:0.5, 0.05×10^6 cells per well for 1:0.25, and 0.02×10^6 cells per well for 1:0.10. The cells were cocultured for 3 days in the presence of soluble mouse anti-CD3 and anti-CD28 antibodies (1 μg/ml each), except for the negative control group. A positive control group was included to assess T cell proliferation in the absence of iT_{regs}. On day 3 of coculture, the supernatant was collected by centrifuging the 96-well plate at 300*g* for 10 min and stored at −80°C until used for measuring IFN-γ levels by enzyme-linked immunosorbent assay (ELISA). The cell pellet was dissolved in PBS and subsequently assessed for CD8$^+$ T cell proliferation by measuring CTV dilution using flow cytometry. For suppressive assay with human iT_{regs}, freshly isolated naïve T cells were labeled with CTV and cocultured with scrambled or CD38 shRNA transduced iT_{regs}.

siRNA-mediated knockdown

Freshly isolated murine naïve CD4$^+$ T cells were differentiated to iT_{regs}, and CD38^{Hi} iT_{regs} were sorted using FACS. To achieve the knockdown of PC/PEPCK, CD38^{Hi} iT_{regs} were electroporated with 150 nM PC/PEPCK-targeting small interfering RNA (siRNA) (MISSION esiRNA; Sigma-Aldrich). Following electroporation, cells were cultured for 48 hours before functional studies were conducted. Statistical validation of functional differences was performed in experiments that achieved at least a 40% reduction in PC/PEPCK expression at the transcript level.

Lentiviral transduction of T cells

Human embryonic kidney (HEK) 293T cells were cultured in T75 flasks before transfection. HEK293T cells were transfected with lentiviral plasmid DNA encoding CD38 shRNA (Origene) or scrambled negative shRNA (Origene) along with structural plasmid ps-PAX2 and p-MD2G using $CaCl_2$ and HBS method. After 24 hours, the medium was replaced with low-serum–containing medium. Four hours after medium change, sodium butyrate was added at 5 mM concentration, and the cells were incubated for additional 48 to 72 hours, after which lentivirus-containing supernatant was collected and filtered. The viral content was concentrated using LentiX Concentrator (Takara Bio, Shinga, Japan) following the manufacturer's protocol and stored as aliquots at −80°C until use. After 3 days of differentiation, human iT_{regs} were collected, washed, and transferred onto non–tissue culture–treated 24-well plates (Corning, NY, USA) coated overnight with RetroNectin (30 μg/ml) (Takara Bio). Cells were transduced with concentrated CD38 shRNA lentiviral/control shRNA lentivirus particle and spinoculated at 1000*g* for 2 hours at 32°C, and IL-2 was added post-spinoculation, followed by a 48-hour culture at 37°C in a CO_2 incubator.

Flow cytometry

Surface staining was performed by incubating cells with the antibody at a 1:200 dilution in FACS buffer (0.5% bovine serum albumin and 0.1% sodium azide in PBS) for 20 min on ice. For intracellular cytokine staining, T cells were restimulated for 4 hours with phorbol 12-myristate 13-acetate (100 ng/ml), ionomycin (1 μg/ml), and GolgiPlug (BD Biosciences, San Jose, CA), followed by staining of surface markers. Cells were then fixed and permeabilized using the BD Cytofix/Cytoperm Kit (BD Biosciences, San Jose, CA), following the manufacturer's protocol. Intracellular cytokine staining was done in the fixed/permeabilized cells. For FoxP3 staining, cells were first stained with surface markers, then fixed and permeabilized using a FoxP3 staining buffer set (Thermo Fisher Scientific, Waltham, MA), and subsequently used for FoxP3 staining. To assess BrdU incorporation, cells were fixed with the FoxP3 staining buffer set, and BrdU staining was performed following deoxyribonuclease treatment.

For all samples, cells were incubated with a fixable live/dead stain (Live/Dead fixable yellow dead cell stain kit from Thermo Fisher Scientific, Waltham, MA) after surface staining, before proceeding with further analysis. All stained samples were acquired using the BD LSRFortessa (BD Biosciences, San Jose, CA) and analyzed with FlowJo software (BD Biosciences). Details of antibodies are provided in table S1.

qPCR analysis

For real-time quantitative polymerase chain reaction (qPCR) analysis, cells were lysed using TRIzol reagent (Thermo Fisher Scientific, Waltham, MA), and RNA was isolated following the TRIzol extraction protocol. The isolated RNA was resuspended in UltraPure water (Thermo Fisher Scientific, Waltham, MA) and quantified using a Biotek Synergy HT Plate Reader. From 1 μg of RNA, cDNA was synthesized using the iScript cDNA Synthesis Kit (Bio-Rad, USA) and diluted 1:3 in nuclease-free water for qPCR reactions. qPCR was performed with the iTaq Universal SYBR Green Supermix (Bio-Rad, USA) on a CFX96 Real-Time System (Bio-Rad, USA) for a total of 40 cycles at a standard reaction speed. The primer details are provided in table S2.

Lactate uptake assay and intracellular NAD^+ assay

Lactate uptake was measured using the Lactate-Glo assay kit (Promega, USA) in 1.5×10^6 cells per group following the manufacturer's protocol. Luminescence was quantified using the Varioskan LUX Multimode Reader (Thermo Fisher Scientific, USA).

Intracellular NAD^+ content was determined in 1×10^6 cells per group using the NAD/NADH Cell-Based Assay Kit (Cayman Chemical, USA). Absorbance at 450 nm was measured using MULTISKAN SkyHigh (Thermo Fisher Scientific, USA).

Enzyme-linked immunosorbent assay

IFN-γ levels in the culture supernatant were measured using the mouse IFN-γ–uncoated ELISA kit (Thermo Fisher Scientific, USA) according to the manufacturer's protocol.

Metabolic flux assay

Metabolic flux analysis was performed using the Seahorse XFe24 analyzer (Agilent Technologies). Briefly, 0.3×10^6 cells were plated on a Seahorse culture plate coated with Cell-Tak (Corning) overnight and incubated at 37°C in a CO_2-free incubator for 30 min to allow the cells to adhere. To assess glycolysis, the ECAR was measured under basal conditions and in response to glucose (10 mM; Sigma Aldrich), oligomycin (1.0 μM; Cayman), and 2-DG (100 mM; Sigma Aldrich). To assess OXPHOS, OCR was measured under basal conditions and in response to oligomycin (1 μM), carbonyl cyanide *p*-trifluoromethoxyphenylhydrazone (FCCP; 1 μM), and rotenone plus antimycin A (2 μM and 100 nM, respectively). Some experiments were conducted with minor modifications; OCR/ECAR analysis was performed under basal conditions or in response to NMN/vehicle control, followed by oligomycin (1 μM), FCCP (1 μM), and rotenone and antimycin A (2 μM and 100 nM).

Metabolomics and stable isotope tracing

For the metabolomics study, 3×10^6 cells were taken, and, before statistical analysis, samples were normalized to Bradford values. Metabolic quenching and extraction of metabolites were performed by using ice-cold 80% methanol, followed by four freeze-thaw cycles in dry ice. Cellular debris was removed by centrifugation at 14,000 rpm for 15 min at 4°C. The supernatant was then collected and stored at −80°C. All the samples were evaporated in nitrogen stream within a hood. Evaporated samples were dissolved in mass spectrometry (MS)–grade water and placed in an autosampler of Ultimate 3000 UHPLC by Thermo Fisher Scientific interfaced to LTQ-Orbitrap XL Mass Spectrometer and the injection amount was 10 μl. The electrospray ionization (ESI)–MS analysis was performed in both negative and positive modes. Full-scan mass spectra were acquired over a mass range of mass/charge ratio of 80 to 1000 at 60,000 resolution, source voltage of 4.5 KV, capillary temperature of 320, and sheath gas flow of 25. Liquid chromatography separation was done in a reverse-phase C18 column (hypersil gold, diameter of 100 mm by 2.1 mm, and particle size of 1.9 μ), maintained at 400°C. Integrated pick areas were then extracted manually using Qual Browser (Thermo Fisher Scientific Xcalibur 2.1).

For isotopic flux analysis, iT_{regs} was generated and, on day 3, FACS sorted into $CD38^{Hi}$ and $CD38^{Lo}$ iT_{regs}. Cells were washed with PBS twice to remove the remaining medium and cultured (2×10^6 cells per well) with glucose-free RPMI 1640 medium supplemented with uniformly labeled ^{13}C L-lactate (Cambridge Isotope Laboratories) for 18 hours. Cells were then washed with cold PBS before

metabolic quenching and extraction using the method described above. Metabolic flux analysis was performed through comprehensive isotopic targeted MS as previously described (*44*). Samples were analyzed on a triple quadrupole hybrid ion trap mass spectrometer (QTRAP 6500+, SCIEX) coupled with a SCIEX ExionLC UHPLC system. Optimized source and gas parameters were for the mass spectrometer, and the data were acquired through Analyst 1.6.3 software in both negative and positive modes. Metabolites (10 μl) were loaded and resolved on an Acquity UPLC BEH HILIC (1.7 μm, 2.1 mm by 100 mm) column where the autosampler temperature was set at 4°C, and the column compartment was set to 40°C. Mobile phases of solvents A (10 mM ammonium acetate and 10 mM ammonium hydroxide in 95% H_2O/5% ACN, pH 9) and B (10 mM ammonium acetate and 10 mM ammonium hydroxide in 5% H_2O/95% ACN, pH 7) were used for separation with a flow rate was 0.3 ml/min. The gradient program used is as follows: 90% buffer B for an initial 1 min, and buffer B was brought to 40% in the next 10 min and held constant for the next 4 min. Buffer B was brought to the initial 90% buffer B concentration in 1 min, and the chromatography system was re-equilibrated for 2 min at this concentration before the next injection. Peak review and area extraction were performed using MultiQuantTM software v.3.0 (SCIEX). A pooled QC from samples was used to analyze the technical variability, and only metabolites with <30% coefficient of variation were quantified.

Immunoblotting

To evaluate the expression of mitochondrial complexes, $CD38^{Hi}$ iT_{regs} and $CD38^{Lo}$ iT_{regs} were lysed using radioimmunoprecipitation assay buffer containing a protease inhibitor cocktail (Thermo Fisher Scientific). Total protein was isolated and quantified using the Bradford assay. Equal amounts of protein (60 μg) were separated by 12% SDS–polyacrylamide gel electrophoresis and transferred to a polyvinylidene difluoride membrane. The membranes were probed overnight at 4°C with an anti-OXPHOS complex antibody (Abcam) at a 1:2000 dilution, followed by incubation with a secondary antibody (horseradish peroxidase–conjugated goat anti-mouse immunoglobulin G at a 1:2000 dilution; Jackson ImmunoResearch Laboratories) for 2 hours at room temperature. Pre-stained protein markers (Invitrogen) were run in parallel to determine the molecular weight of the proteins. For chemiluminescent detection, the membranes were treated with Clarity Western ECL substrate (Bio-Rad), and the signal was monitored using the Bio-Rad Versa Doc Imaging System (Bio-Rad). The same membrane was then stripped with stripping buffer and re-probed with an antibody specific to heat shock protein 60 (HSP 60) (BioBharati LifeScience) as a reference control.

Single-cell sequencing data analyses

In the scRNA-seq analysis using Seurat (v4.3.0), the gene expression matrices for each sample were imported and quality controlled by filtering cells on the basis of mitochondrial gene expression and feature counts. The data were then normalized using the "LogNormalize" method that normalizes feature expression by scaling each cell's measurements on the basis of the total expression and then applies a natural logarithm transformation. Next, we identified most variable genes per sample with "FindVariableFeatures" using the "vst" method. Further, the gene expression matrices were merged into a single matrix and linearly transformed with "ScaleData" function, and, subsequently, dimensionality reduction was done with "RunPCA" function in Seurat, capturing the main sources of variation in the dataset. To address batch effects, the scaled principal components analysis matrix was processed with the "RunHarmony" function (v1.0) in Seurat, integrating the data to minimize technical variation and enable unsupervised clustering. *t*–Distributed stochastic neighbor embedding plots of the harmony-corrected embeddings visualized cell clusters. Cluster analysis used a shared nearest neighbor graph and the Louvain algorithm to group cells by gene expression patterns. Marker genes for each cluster were identified with "FindAllMarkers" and visualized using heatmaps and feature plots created with ggplot2, providing insights into the gene expression profiles of different cell types or states.

DNA methylation assay

For the methylation study, 1×10^6 cells per replicate were used to examine specific methylation in the CNS1, CNS2, and CNS3 regions of the *Foxp3* gene. The study involved bisulfite conversion of genomic DNA followed by PCR amplification. Bisulfite treatment converts cytosine residues to uracil while leaving 5-methylcytosine intact, using the Methylamp DNA Modification Kit. Approximately 60 ng of DNA was used for methylation analysis, which was performed by qPCR with the Methylamp MS q-PCR Fast Kit (Epigentec). The study was conducted according to the manufacturer's protocol.

Mouse tumor experiment

B16-F10 melanoma (0.5×10^6 cells per mouse), YUMM1.7 melanoma (0.75×10^6 cells per mouse), or EL4 thymoma (1×10^6 cells per mouse) was injected subcutaneously into the flanks of 6- to 8-week-old C57BL/6 mice. Tumor volumes were measured every 2 days following tumor engraftment. For the CD38i experiment, YUMM1.7 melanoma-bearing mice were administered either CD38i (15 mg/kg) or vehicle control intraperitoneally on day 6 post–tumor inoculation. CD38i was then given intraperitoneally once a week until the tumor volume in control mice reached a humane endpoint. To assess intratumoral T_{regs} proliferation, 150 μl (150 mg) of BrdU solution (BioLegend) was injected intraperitoneally into both control and CD38i-treated YUMM1.7 melanoma-bearing mice 1 day before euthanasia.

Statistical analysis

For statistical analysis between two groups, the nonparametric unpaired Wilcoxon-Mann-Whitney *U* test was applied. Comparisons involving more than two groups were performed using one-way or two-way analysis of variance (ANOVA). Data analysis was conducted using GraphPad Prism 9.

Supplementary Materials

This PDF file includes:
Figs. S1 to S6
Tables S1 and S2

REFERENCES AND NOTES

1. T. Kamada, Y. Togashi, C. Tay, D. Ha, A. Sasaki, Y. Nakamura, E. Sato, S. Fukuoka, Y. Tada, A. Tanaka, H. Morikawa, A. Kawazoe, T. Kinoshita, K. Shitara, S. Sakaguchi, H. Nishikawa, $PD\text{-}1^+$ regulatory T cells amplified by PD-1 blockade promote hyperprogression of cancer. *Proc. Natl. Acad. Sci. U.S.A.* **116**, 9999–10008 (2019).
2. R. P. M. Sutmuller, L. M. van Duivenvoorde, A. van Elsas, T. N. M. Schumacher, M. E. Wildenberg, J. P. Allison, R. E. M. Toes, R. Offringa, C. J. M. Melief, Synergism of cytotoxic T lymphocyte–associated antigen 4 blockade and depletion of $CD25^+$ regulatory T cells in antitumor therapy reveals alternative pathways for suppression of autoreactive cytotoxic T lymphocyte responses. *J. Exp. Med.* **194**, 823–832 (2001).

3. A. Tanaka, S. Sakaguchi, Regulatory T cells in cancer immunotherapy. *Cell Res.* **27**, 109–118 (2017).
4. Y. Togashi, K. Shitara, H. Nishikawa, Regulatory T cells in cancer immunosuppression - implications for anticancer therapy. *Nat. Rev. Clin. Oncol.* **16**, 356–371 (2019).
5. S. Sakaguchi, Naturally arising $CD4^+$ regulatory t cells for immunologic self-tolerance and negative control of immune responses. *Annu. Rev. Immunol.* **22**, 531–562 (2004).
6. N. M. Chapman, H. Chi, Metabolic adaptation of lymphocytes in immunity and disease. *Immunity* **55**, 14–30 (2022).
7. A. Angelin, L. Gil-de-Gomez, S. Dahiya, J. Jiao, L. Guo, M. H. Levine, Z. Wang, W. J. Quinn III, P. K. Kopinski, L. Wang, T. Akimova, Y. Liu, T. R. Bhatti, R. Han, B. L. Laskin, J. A. Baur, I. A. Blair, D. C. Wallace, W. W. Hancock, U. H. Beier, Foxp3 reprograms T cell metabolism to function in low-glucose, high-lactate environments. *Cell Metab.* **25**, 1282–1293.e7 (2017).
8. M. J. Watson, P. D. A. Vignali, S. J. Mullett, A. E. Overacre-Delgoffe, R. M. Peralta, S. Grebinoski, A. V. Menk, N. L. Rittenhouse, K. DePeaux, R. D. Whetstone, D. A. A. Vignali, T. W. Hand, A. C. Poholek, B. M. Morrison, J. D. Rothstein, S. G. Wendell, G. M. Delgoffe, Metabolic support of tumour-infiltrating regulatory T cells by lactic acid. *Nature* **591**, 645–651 (2021).
9. C. S. Field, F. Baixauli, R. L. Kyle, D. J. Puleston, A. M. Cameron, D. E. Sanin, K. L. Hippen, M. Loschi, G. Thangavelu, M. Corrado, J. Edwards-Hicks, K. M. Grzes, E. J. Pearce, B. R. Blazar, E. L. Pearce, Mitochondrial integrity regulated by lipid metabolism is a cell-intrinsic checkpoint for Treg suppressive function. *Cell Metab.* **31**, 422–437.e5 (2020).
10. H. Wang, F. Franco, Y. C. Tsui, X. Xie, M. P. Trefny, R. Zappasodi, S. R. Mohmood, J. Fernandez-Garcia, C. H. Tsai, I. Schulze, F. Picard, E. Meylan, R. Silverstein, I. Goldberg, S. M. Fendt, J. D. Wolchok, T. Merghoub, C. Jandus, A. Zippelius, P. C. Ho, CD36-mediated metabolic adaptation supports regulatory T cell survival and function in tumors. *Nat. Immunol.* **21**, 298–308 (2020).
11. M. I. Matias, C. S. Yong, A. Foroushani, C. Goldsmith, C. Mongellaz, E. Sezgin, K. R. Levental, A. Talebi, J. Perrault, A. Riviere, J. Dehairs, O. Delos, J. Bertand-Michel, J.-C. Portais, M. Wong, J. C. Marie, A. Kelekar, S. Kinet, V. S. Zimmermann, I. Levental, L. Yvan-Charvet, J. V. Swinnen, S. A. Muljo, H. Hernandez-Vargas, S. Tardito, N. Taylor, V. Dardalhon, Regulatory T cell differentiation is controlled by αKG-induced alterations in mitochondrial metabolism and lipid homeostasis. *Cell Rep.* **37**, 109911 (2021).
12. A. Brand, K. Singer, G. E. Koehl, M. Kolitzus, G. Schoenhammer, A. Thiel, C. Matos, C. Bruss, S. Klobuch, K. Peter, M. Kastenberger, C. Bogdan, U. Schleicher, A. Mackensen, E. Ullrich, S. Fichtner-Feigl, R. Kesselring, M. Mack, U. Ritter, M. Schmid, C. Blank, K. Dettmer, P. J. Oefner, P. Hoffmann, S. Walenta, E. K. Geissler, J. Pouyssegur, A. Villunger, A. Steven, B. Seliger, S. Schreml, S. Haferkamp, E. Kohl, S. Karrer, M. Berneburg, W. Herr, W. Mueller-Klieser, K. Renner, M. Kreutz, LDHA-associated lactic acid production blunts tumor immunosurveillance by T and NK cells. *Cell Metab.* **24**, 657–671 (2016).
13. R. Haas, J. Smith, V. Rocher-Ros, S. Nadkarni, T. Montero-Melendez, F. D'Acquisto, E. J. Bland, M. Bombardieri, C. Pitzalis, M. Perretti, F. M. Marelli-Berg, C. Mauro, Lactate regulates metabolic and pro-inflammatory circuits in control of T cell migration and effector functions. *PLOS Biol.* **13**, e1002202 (2015).
14. S. Jitrapakdee, M. St Maurice, I. Rayment, W. W. Cleland, J. C. Wallace, P. V. Attwood, Structure, mechanism and regulation of pyruvate carboxylase. *Biochem. J.* **413**, 369–387 (2008).
15. M. Holecek, Roles of malate and aspartate in gluconeogenesis in various physiological and pathological states. *Metabolism* **145**, 155614 (2023).
16. D. Klysz, X. Tai, P. A. Robert, M. Craveiro, G. Cretenet, L. Oburoglu, C. Mongellaz, S. Floess, V. Fritz, M. I. Matias, C. Yong, N. Surh, J. C. Marie, J. Huehn, V. Zimmermann, S. Kinet, V. Dardalhon, N. Taylor, Glutamine-dependent alpha-ketoglutarate production regulates the balance between T helper 1 cell and regulatory T cell generation. *Sci. Signal.* **8**, ra97 (2015).
17. A. Kar, P. Ghosh, A. Gautam, S. Chowdhury, D. Basak, I. Sarkar, A. Bhoumik, S. Barman, P. Chakraborty, A. Mukhopadhyay, S. Mehrotra, S. K. Ganesan, S. Paul, S. Chatterjee, CD38-RyR2 axis-mediated signaling impedes $CD8^+$ T cell response to anti-PD1 therapy in cancer. *Proc. Natl. Acad. Sci. U.S.A.* **121**, e2315989121 (2024).
18. Y. Huang, M. Shao, X. Teng, X. Si, L. Wu, P. Jiang, L. Liu, B. Cai, X. Wang, Y. Han, Y. Feng, K. Liu, Z. Zhang, J. Cui, M. Zhang, Y. Hu, P. Qian, H. Huang, Inhibition of CD38 enzymatic activity enhances CAR-T cell immune-therapeutic efficacy by repressing glycolytic metabolism. *Cell Rep. Med.* **5**, 101400 (2024).
19. P. M. Chen, E. Katsuyama, A. Satyam, H. Li, J. Rubio, S. Jung, S. Andrzejewski, J. D. Becherer, M. G. Tsokos, R. Abdi, G. C. Tsokos, CD38 reduces mitochondrial fitness and cytotoxic T cell response against viral infection in lupus patients by suppressing mitophagy. *Sci. Adv.* **8**, eabo4271 (2022).
20. S. Chatterjee, A. Daenthanasanmak, P. Chakraborty, M. W. Wyatt, P. Dhar, S. P. Selvam, J. Fu, J. Zhang, H. Nguyen, I. Kang, K. Toth, M. Al-Homrani, M. Husain, G. Beeson, L. Ball, K. Helke, S. Husain, E. Garrett-Mayer, G. Hardiman, M. Mehrotra, M. I. Nishimura, C. C. Beeson, M. G. Bupp, J. Wu, B. Ogretmen, C. M. Paulos, J. Rathmell, X. Z. Yu, S. Mehrotra, CD38-NAD^+ axis regulates immunotherapeutic anti-tumor T cell response. *Cell Metab.* **27**, 85–100.e8 (2018).
21. J. Krejcik, T. Casneuf, I. S. Nijhof, B. Verbist, J. Bald, T. Plesner, K. Syed, K. Liu, N. W. C. J. van de Donk, B. M. Weiss, T. Ahmadi, H. M. Lokhorst, T. Mutis, A. K. Sasser, Daratumumab depletes $CD38^+$ immune regulatory cells, promotes T-cell expansion, and skews T-cell repertoire in multiple myeloma. *Blood* **128**, 384–394 (2016).
22. X. Feng, L. Zhang, C. Acharya, G. An, K. Wen, L. Qiu, N. C. Munshi, Y. T. Tai, K. C. Anderson, Targeting CD38 suppresses induction and function of T regulatory cells to mitigate immunosuppression in multiple myeloma. *Clin. Cancer Res.* **23**, 4290–4300 (2017).
23. C. Tay, A. Tanaka, S. Sakaguchi, Tumor-infiltrating regulatory T cells as targets of cancer immunotherapy. *Cancer Cell* **41**, 450–465 (2023).
24. T. Saito, H. Nishikawa, H. Wada, Y. Nagano, D. Sugiyama, K. Atarashi, Y. Maeda, M. Hamaguchi, N. Ohkura, E. Sato, H. Nagase, J. Nishimura, H. Yamamoto, S. Takiguchi, T. Tanoue, W. Suda, H. Morita, M. Hattori, K. Honda, M. Mori, Y. Doki, S. Sakaguchi, Two $FOXP3^+CD4^+$ T cell subpopulations distinctly control the prognosis of colorectal cancers. *Nat. Med.* **22**, 679–684 (2016).
25. E. Azizi, A. J. Carr, G. Plitas, A. E. Cornish, C. Konopacki, S. Prabhakaran, J. Nainys, K. Wu, V. Kiseliovas, M. Setty, K. Choi, R. M. Fromme, P. Dao, P. T. McKenney, R. C. Wasti, K. Kadaveru, L. Mazutis, A. Y. Rudensky, D. Pe'er, Single-cell map of diverse immune phenotypes in the breast tumor microenvironment. *Cell* **174**, 1293–1308.e36 (2018).
26. M. F. du Pre, L. A. van Berkel, M. Raki, M. A. van Leeuwen, L. F. de Ruiter, F. Broere, M. N. Ter Borg, F. E. Lund, J. C. Escher, K. E. Lundin, L. M. Sollid, G. Kraal, E. E. Nieuwenhuis, J. N. Samsom, $CD62L^{neg}CD38^+$ expression on circulating $CD4^+$ T cells identifies mucosally differentiated cells in protein fed mice and in human celiac disease patients and controls. *Am. J. Gastroenterol.* **106**, 1147–1159 (2011).
27. U. H. Beier, A. Angelin, T. Akimova, L. Wang, Y. Liu, H. Xiao, M. A. Koike, S. A. Hancock, T. R. Bhatti, R. Han, J. Jiao, S. C. Veasey, C. A. Sims, J. A. Baur, D. C. Wallace, W. W. Hancock, Essential role of mitochondrial energy metabolism in $Foxp3^+$ T-regulatory cell function and allograft survival. *FASEB J.* **29**, 2315–2326 (2015).
28. S. E. Weinberg, B. D. Singer, E. M. Steinert, C. A. Martinez, M. M. Mehta, I. Martinez-Reyes, P. Gao, K. A. Helmin, H. Abdala-Valencia, L. A. Sena, P. T. Schumacker, L. A. Turka, N. S. Chandel, Mitochondrial complex III is essential for suppressive function of regulatory T cells. *Nature* **565**, 495–499 (2019).
29. C. C. S. Chini, T. R. Peclat, G. M. Warner, S. Kashyap, J. M. Espindola-Netto, G. C. de Oliveira, L. S. Gomez, K. A. Hogan, M. G. Tarrago, A. S. Puranik, G. Agorrody, K. L. Thompson, K. Dang, S. Clarke, B. G. Childs, K. S. Kanamori, M. A. Witte, P. Vidal, A. L. Kirkland, M. De Cecco, K. Chellappa, M. R. McReynolds, C. Jankowski, T. Tchkonia, J. L. Kirkland, J. M. Sedivy, J. M. van Deursen, D. J. Baker, W. van Schooten, J. D. Rabinowitz, J. A. Baur, E. N. Chini, CD38 ecto-enzyme in immune cells is induced during aging and regulates NAD^+ and NMN levels. *Nat. Metab.* **2**, 1284–1304 (2020).
30. Y. Yang, A. A. Sauve, NAD^+ metabolism: Bioenergetics, signaling and manipulation for therapy. *Biochim. Biophys. Acta* **1864**, 1787–1800 (2016).
31. Y. Zheng, S. Josefowicz, A. Chaudhry, X. P. Peng, K. Forbush, A. Y. Rudensky, Role of conserved non-coding DNA elements in the Foxp3 gene in regulatory T-cell fate. *Nature* **463**, 808–812 (2010).
32. S. Floess, J. Freyer, C. Siewert, U. Baron, S. Olek, J. Polansky, K. Schlawe, H. D. Chang, T. Bopp, E. Schmitt, S. Klein-Hessling, E. Serfling, A. Hamann, J. Huehn, Epigenetic control of the foxp3 locus in regulatory T cells. *PLOS Biol.* **5**, e38 (2007).
33. Y. Feng, A. Arvey, T. Chinen, J. van der Veeken, G. Gasteiger, A. Y. Rudensky, Control of the inheritance of regulatory T cell identity by a *cis* element in the *Foxp3* locus. *Cell* **158**, 749–763 (2014).
34. R. Kawakami, Y. Kitagawa, K. Y. Chen, M. Arai, D. Ohara, Y. Nakamura, K. Yasuda, M. Osaki, N. Mikami, C. A. Lareau, H. Watanabe, G. Kondoh, K. Hirota, N. Ohkura, S. Sakaguchi, Distinct Foxp3 enhancer elements coordinate development, maintenance, and function of regulatory T cells. *Immunity* **54**, 947–961.e8 (2021).
35. A. Facciabene, X. Peng, I. S. Hagemann, K. Balint, A. Barchetti, L. P. Wang, P. A. Gimotty, C. B. Gilks, P. Lal, L. Zhang, G. Coukos, Tumour hypoxia promotes tolerance and angiogenesis via CCL28 and T_{reg} cells. *Nature* **475**, 226–230 (2011).
36. S. Suthen, C. J. Lim, P. H. D. Nguyen, C. A. Dutertre, H. L. H. Lai, M. Wasser, C. Chua, T. K. H. Lim, W. Q. Leow, T. J. Loh, W. K. Wan, Y. H. Pang, G. Soon, P. C. Cheow, J. H. Kam, S. Iyer, A. Kow, W. L. Tam, T. W. H. Shuen, H. C. Toh, Y. Y. Dan, G. K. Bonney, C. Y. Chan, A. Chung, B. K. P. Goh, W. Zhai, F. Ginhoux, P. K. H. Chow, S. Albani, V. Chew, Hypoxia-driven immunosuppression by Treg and type-2 conventional dendritic cells in HCC. *Hepatology* **76**, 1329–1344 (2022).
37. V. A. Gerriets, R. J. Kishton, M. O. Johnson, S. Cohen, P. J. Siska, A. G. Nichols, M. O. Warmoes, A. A. de Cubas, N. J. MacIver, J. W. Locasale, L. A. Turka, A. D. Wells, J. C. Rathmell, Foxp3 and Toll-like receptor signaling balance T_{reg} cell anabolic metabolism for suppression. *Nat. Immunol.* **17**, 1459–1466 (2016).
38. Y. J. Zhao, C. M. Lam, H. C. Lee, The membrane-bound enzyme CD38 exists in two opposing orientations. *Sci. Signal.* **5**, ra67 (2012).
39. J. Liu, Y. J. Zhao, W. H. Li, Y. N. Hou, T. Li, Z. Y. Zhao, C. Fang, S. L. Li, H. C. Lee, Cytosolic interaction of type III human CD38 with CIB1 modulates cellular cyclic ADP-ribose levels. *Proc. Natl. Acad. Sci. U.S.A.* **114**, 8283–8288 (2017).

40. A. Kar, S. Mehrotra, S. Chatterjee, CD38: T cell immuno-metabolic modulator. *Cells* **9**, 1716 (2020).
41. C. Procaccini, F. Carbone, D. Di Silvestre, F. Brambilla, V. De Rosa, M. Galgani, D. Faicchia, G. Marone, D. Tramontano, M. Corona, C. Alviggi, A. Porcellini, A. La Cava, P. Mauri, G. Matarese, The proteomic landscape of human ex vivo regulatory and conventional T cells reveals specific metabolic requirements. *Immunity* **44**, 406–421 (2016).
42. H. Kurniawan, L. Soriano-Baguet, D. Brenner, Regulatory T cell metabolism at the intersection between autoimmune diseases and cancer. *Eur. J. Immunol.* **50**, 1626–1642 (2020).
43. T. A. Karakasheva, T. J. Waldron, E. Eruslanov, S. B. Kim, J. S. Lee, S. O'Brien, P. D. Hicks, D. Basu, S. Singhal, F. Malavasi, A. K. Rustgi, CD38-expressing myeloid-derived suppressor cells promote tumor growth in a murine model of esophageal cancer. *Cancer Res.* **75**, 4074–4085 (2015).
44. X. Shi, B. Xi, P. Jasbi, C. Turner, Y. Jin, H. Gu, Comprehensive isotopic targeted mass spectrometry: Reliable metabolic flux analysis with broad coverage. *Anal. Chem.* **92**, 11728–11738 (2020).

Acknowledgments

Funding: This work was supported by the DBT/Wellcome Trust India Alliance Intermediate Fellowship Grant IA/I/19/1/504277. S.Cha. also acknowledges funding from the Council of Scientific and Industrial Research (CSIR), Government of India (HCP-43), as well as P50 and MLP-121 funds from CSIR-IICB, Kolkata, and support from the Central Instrumentation Facility (CIF) of CSIR-IICB, Kolkata. A.G. acknowledges support from the High Performance and Cloud Computing Group at the Zentrum für Datenverarbeitung of the University of Tübingen, the state of Baden-Württemberg through bwHPC, and the German Research Foundation (DFG) through grant no. INST 37/935-1 FUGG. A.G. also receives support from the de.NBI Cloud within the German Network for Bioinformatics Infrastructure (de.NBI) and ELIXIR-DE (Forschungszentrum Jülich and W-de.NBI-001, W-de.NBI-004, W-de.NBI-008, W-de.NBI-010, W-de.NBI-013, W-de.NBI-014, W-de.NBI-016, W-de.NBI-022) to conduct computational analysis in this work. **Author contributions:** Conceptualization: I.S. and S. Cha. Methodology: I.S., P.S., L.C., S.Maj., S.P., S.S., and S.Cha. Investigation: I.S., D.B., A.B., P.S., A.K., S.Mah., S.Cho., L.C., and S.P. Visualization: P.G., S.Mo., R.M., S.P., and S.Cha. Formal analysis: I.S., D.B., P.G., A.G., A.B., P.S., S.Mah., L.C., S.Me., S.Maj., S.S., S.P., and S.Cha. Data curation: S.P. Software: P.G., A.G., and S.P. Resources: P.S., S.Mo., S.Me., R.M., S.S., S.P., and S.Cha. Validation: I.S., D.B., P.G., A.G., A.K., S.Cho., S.S., S.P., and S.Cha. Funding acquisition: S.Cha. Project administration: S.Cha. Supervision: S.S. and S.Cha. Writing—original draft: S.Cha. Writing—review and editing: I.S., D.B., P.G., A.G., P.S., A.K., S.Mah., S.Cho., L.C., S.Me., S.Maj., S.S., S.P., and S.Cha. **Competing interests:** The authors declare that they have no competing interests. **Data and materials availability:** All data needed to evaluate the conclusions in the paper are present in the paper and/or the Supplementary Materials.

Submitted 17 September 2024
Accepted 14 February 2025
Published 21 March 2025
10.1126/sciadv.adt2117
