# Supplementary material for: CD38-mediated metabolic reprogramming promotes the stability and suppressive function of regulatory T cells in tumor
Source: Sci Adv. 2025 Mar 21;11(12):eadt2117. doi: 10.1126/sciadv.adt2117 (PMC11927613; doi:10.1126/sciadv.adt2117)
Supplement: Supplementary file 4 [file sciadv.adt2117_sm.v2.pdf]

ERRATUM PUBLISHED 6 NOVEMBER 2026; SEE BELOW

Science Advances
AAAS

Supplementary Materials for

CD38-mediated metabolic reprogramming promotes the stability and suppressive function of regulatory T cells in tumor

Ishita Sarkar *et al.*

Corresponding author: Shilpak Chatterjee, schatterjee@iicb.res.in

Sci. Adv. **11**, eadt2117 (2025)
DOI: 10.1126/sciadv.adt2117

This PDF file includes:

Figs. S1 to S6
Tables S1 and S2

Erratum (6 November 2026): The original Research Article contained errors in Figs. 4, 6, and fig. S6. Figures 4 and 6 have been replaced with the corrected versions and the Supplementary Materials file has been replaced with the corrected version of fig. S6.

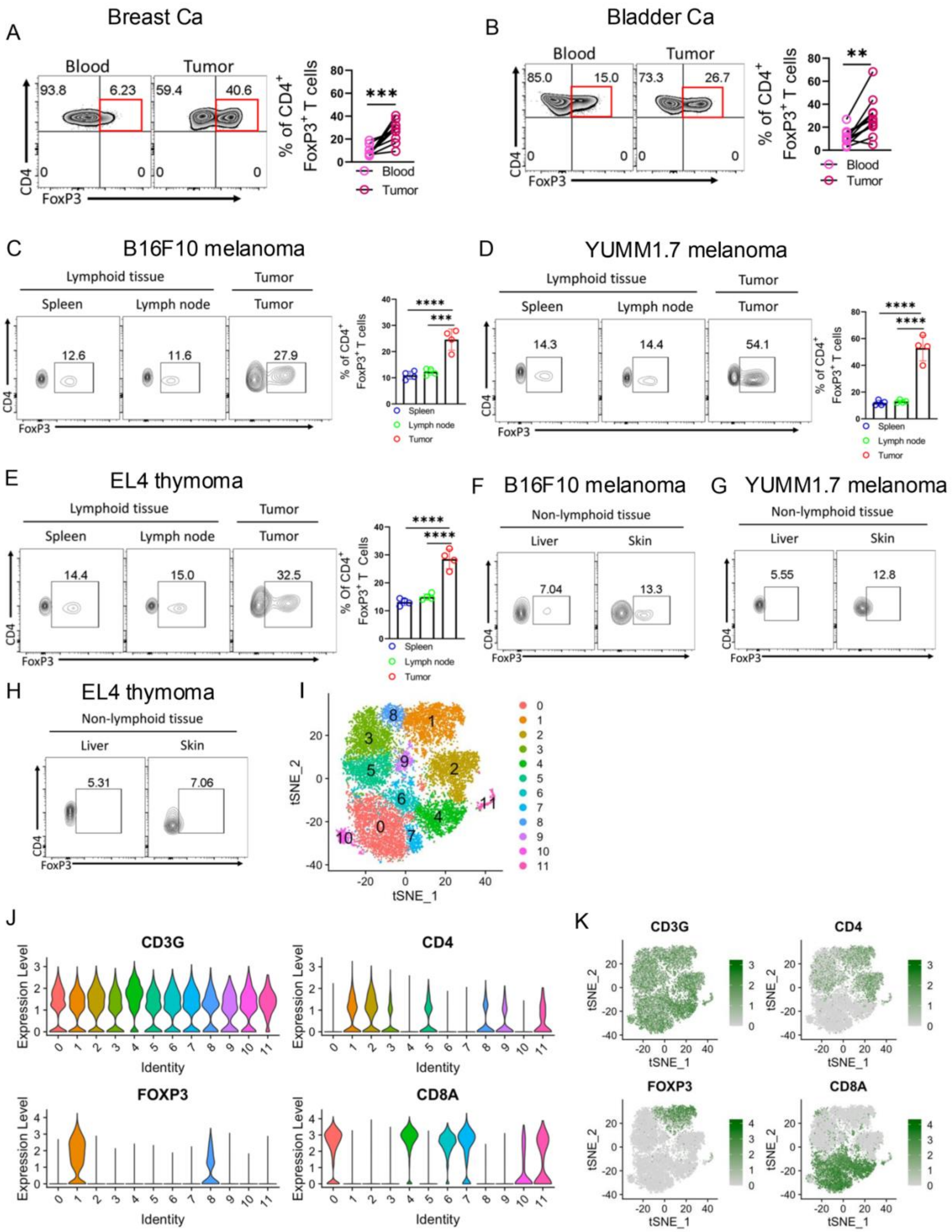

A
Breast Ca
Blood
Tumor
93.8
6.23
59.4
40.6
0
0
0
0
CD4
FoxP3
% of CD4+ FoxP3+ T cells
***
Blood
Tumor
B
Bladder Ca
Blood
Tumor
85.0
15.0
73.3
26.7
**
C
B16F10 melanoma
Lymphoid tissue
Spleen
Lymph node
Tumor
12.6
11.6
27.9
****
***
D
YUMM1.7 melanoma
14.3
14.4
54.1
E
EL4 thymoma
14.4
15.0
32.5
% Of CD4+ FoxP3+ T Cells
F
B16F10 melanoma
Non-lymphoid tissue
Liver
Skin
7.04
13.3
G
YUMM1.7 melanoma
5.55
12.8
H
EL4 thymoma
5.31
7.06
I
tSNE_1
tSNE_2
J
CD3G
CD4
FOXP3
CD8A
Expression Level
Identity
K

Figure S1. Analysis of Tregs at the tumor site. (A-B) The frequency of Tregs ($CD4^+FoxP3^+$ T cells) was determined in: (A-B) tumor tissue and paired blood samples from patients bearing (A) breast cancer and (B) muscle-invasive bladder cancer. Adjacent bar plots represent cumulative data from nine (A) and twelve (B) samples. (C-H) The frequency of Tregs ($CD4^+FoxP3^+$ T cells) was determined in: (C-E) lymphoid tissues (spleen and DLN) and tumor tissue, (F-H) non-lymphoid tissues (liver and skin) from B6 mice bearing 15 days subcutaneously established (C and F) B16-F10 melanoma, (D and G) YUMM1.7 melanoma, and (E and H) EL-4 thymoma. Adjacent bar diagrams represent data from four (C-H) independent experiments. (I) tSNE visualisation of the scRNA-seq clusters of *CD3G*$^+$ T cells from 5 breast cancer samples (GSE114727). (J) Violin plot showing expression of *CD3G* (Pan T cell marker), *CD4* (Marker for $CD4^+$ T cell), *FOXP3* (Master transcription factor for Treg cell), *CD8A* (Marker for $CD8^+$ T cell) genes across the scRNA-seq clusters of $CD3^+$ T cells. (K) Single-cell gene expression levels of representative genes illustrated in the tSNE plot. Gene expressions are color-coded: green-expressed; grey-not expressed. *, $P < 0.05$; **, $P < 0.01$; ***, $P < 0.005$; ****, $P < 0.0001$.

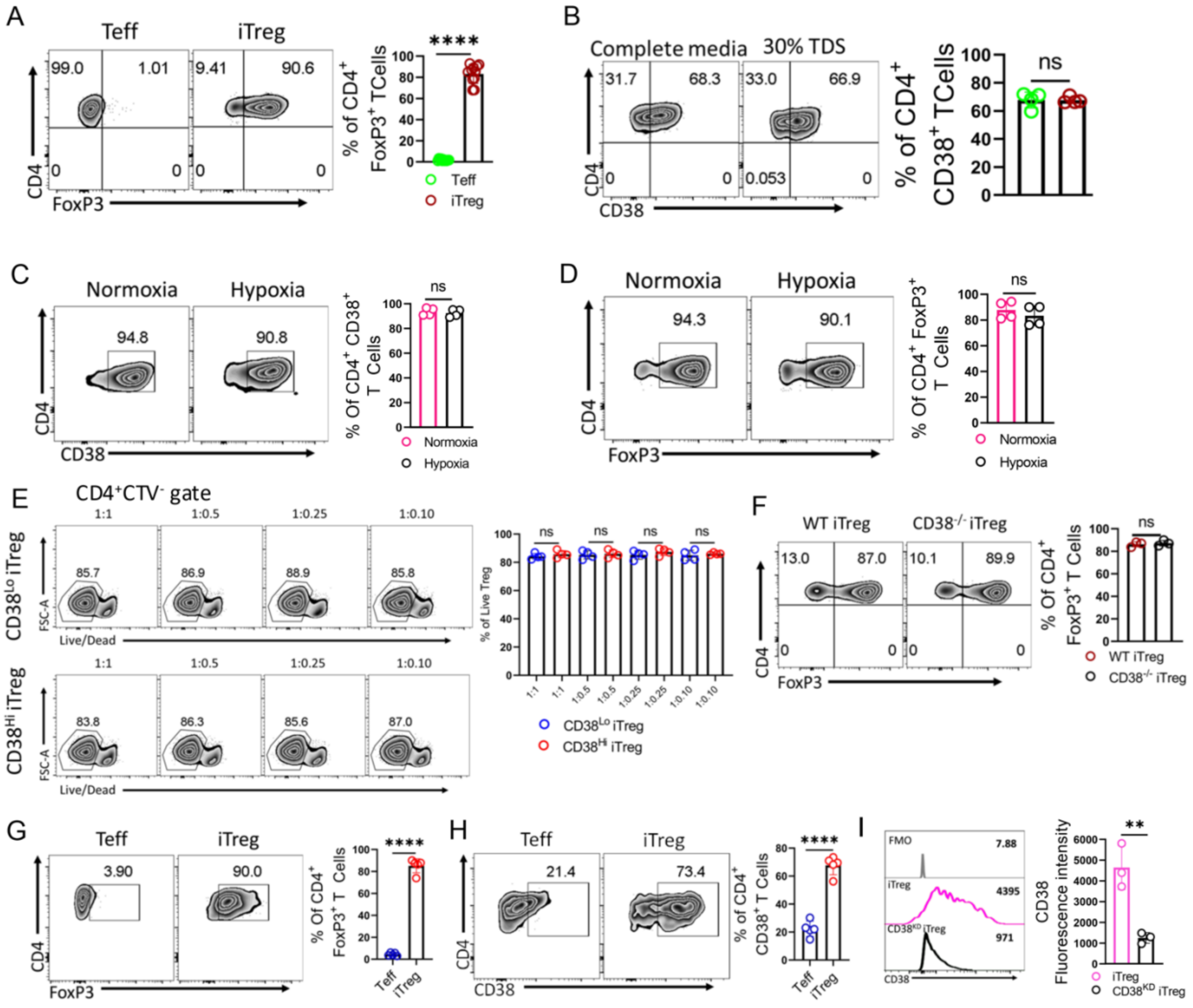

A
Teff
iTreg
99.0
1.01
9.41
90.6
CD4
FoxP3
% of CD4+ FoxP3+ TCells
****
Teff
iTreg
B
Complete media
30% TDS
31.7
68.3
33.0
66.9
0.053
CD38
% of CD4+ CD38+ TCells
ns
C
Normoxia
Hypoxia
94.8
90.8
% Of CD4+ CD38+ T Cells
D
94.3
90.1
% Of CD4+ FoxP3+ T Cells
E
CD4+CTV- gate
1:1
1:0.5
1:0.25
1:0.10
CD38Lo iTreg
CD38Hi iTreg
FSC-A
Live/Dead
85.7
86.9
88.9
85.8
83.8
86.3
85.6
87.0
% of Live Treg
F
WT iTreg
CD38-/- iTreg
13.0
87.0
10.1
89.9
G
3.90
90.0
H
21.4
73.4
I
FMO
7.88
4395
CD38KD iTreg
971
CD38 Fluorescence intensity
**

Figure S2. Characterization of in vitro differentiation of Tregs. (A) The frequency of $CD4^+$ T cells expressing FoxP3 was evaluated in *in vitro* differentiated Teff and Treg populations. (B) Naïve $CD4^+$ T cells were differentiated to iTreg either in complete media or 30% TDS, and the frequency of $CD4^+$ T cells expressing CD38 was evaluated within the Treg compartment ($CD4^+FoxP3^+$ T cells). (C-D) Naïve $CD4^+$ T cells were differentiated to iTreg either in normoxia or hypoxia and the frequency of $CD4^+$ T cells expressing (C) CD38 and (D) FoxP3 were evaluated. (E) Assessing the percentage of viable iTreg cells ($CD4^+CTV^-$ gate) after three days of co-culture with CTV-labeled T cells from WT mice. (F) Naïve $CD4^+$ T cells from WT and $CD38^{-/-}$ mice were differentiated to iTregs and assessed for the frequency of $CD4^+$ T cells expressing FoxP3. (G) Naïve $CD4^+$ T cells isolated from healthy human PBMC were differentiated to either iTreg or Teff cells and assessed for the frequency of $Foxp3^+$ cells within the $CD4^+$ T cell population. (H) Expression of CD38 on Teff and iTregs differentiated from $CD4^+$ T cells. (I) Human iTregs were transduced with either control shRNA or CD38 shRNA and assessed for CD38 expression. Adjacent bar diagrams represent data from eleven (A), four (B-E), three (F), five (G-H), and three (I) independent experiments. *, $P < 0.05$; **, $P < 0.01$; ***, $P < 0.005$; ****, $P < 0.0001$.

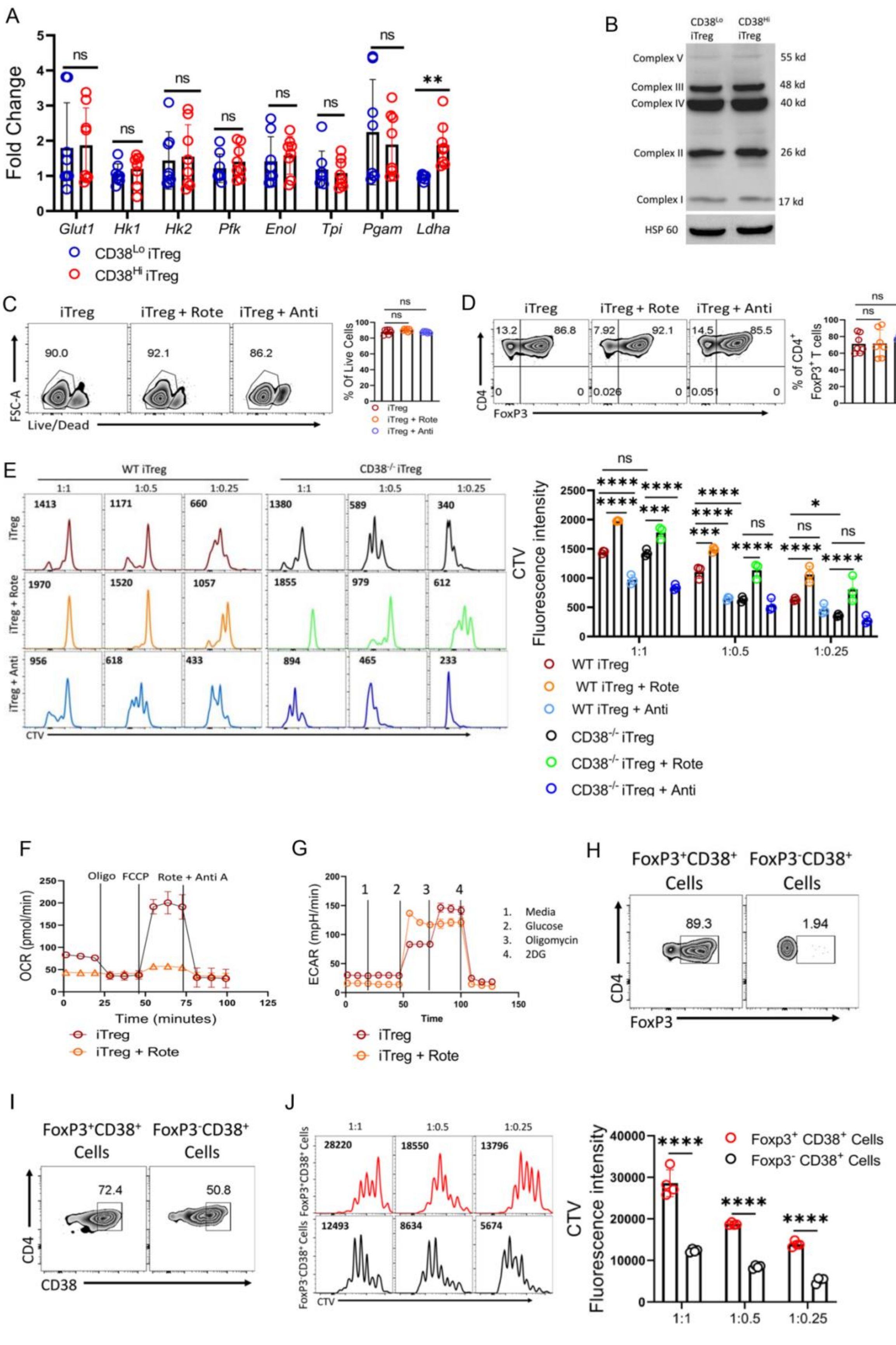

A
Fold Change
ns
**
Glut1
Hk1
Hk2
Pfk
Enol
Tpi
Pgam
Ldha
CD38Lo iTreg
CD38Hi iTreg
B
CD38Lo iTreg
CD38Hi iTreg
Complex V
Complex III
Complex IV
Complex II
Complex I
HSP 60
55 kd
48 kd
40 kd
26 kd
17 kd
C
iTreg
iTreg + Rote
iTreg + Anti
90.0
92.1
86.2
FSC-A
Live/Dead
% Of Live Cells
D
13.2
86.8
7.92
92.1
14.5
85.5
CD4
FoxP3
% of CD4+ FoxP3+ T cells
E
WT iTreg
CD38-/- iTreg
1:1
1:0.5
1:0.25
CTV
CTV Fluorescence intensity
WT iTreg + Rote
WT iTreg + Anti
CD38-/- iTreg + Rote
CD38-/- iTreg + Anti
F
Oligo
FCCP
Rote + Anti A
OCR (pmol/min)
Time (minutes)
G
ECAR (mpH/min)
Time
1. Media
2. Glucose
3. Oligomycin
4. 2DG
H
FoxP3+CD38+ Cells
FoxP3-CD38+ Cells
89.3
1.94
I
72.4
50.8
CD38
J
Foxp3+ CD38+ Cells
Foxp3- CD38+ Cells

Figure S3. Metabolic traits in CD38^{Hi} and CD38^{Lo} iTregs. (A) qPCR analysis of genes in CD38^{Hi} and CD38^{Lo} iTregs. The data are representative of eight independent experiments. (B) Western blot analysis showing the expression of various mitochondrial complexes in FACS-sorted CD38^{Hi} and CD38^{Lo} iTregs, with Hsp60 (a mitochondrial matrix protein) used as a loading control. Data are representative of three independent experiments. (C-D) iTregs differentiated in the presence or absence of indicated inhibitors were used for: (C) Live/dead staining to assess cell viability and (D) determining the frequency of CD4^{+} T cells expressing FoxP3. Adjacent bar diagrams represent data from seven independent experiments. (E) WT or CD38 $^{-/-}$iTregs were differentiated in the presence or absence of rotenone (Rote: 1 μM, added at 48h) and antimycin A (Anti: 1 μM, added at 48h). After washing, they were co-cultured with CTV-labelled T cells in the presence of a T cell activation cocktail (anti-CD3/CD28) for three days. CD8^{+} T cell proliferation was assessed via CTV dilution. The bar diagram (bottom panel) shows data from four independent experiments. (F-G) iTregs differentiated in the presence or absence of rotenone were analyzed for: (F) OCR under basal condition and in response to different indicated inhibitors and (G) ECAR in response to glucose, oligomycin, and 2DG. (H-J) Naïve CD4^{+} T cells were either chronically expanded or differentiated to iTregs and assessed for the frequency of (H) Foxp3 and (I) CD38 expression. (J) FoxP3^{+} CD38^{+} and FoxP3^{-} CD38^{+} cells were co-cultured with CTV-labeled naïve T cells in the presence of anti-CD3/CD28 for three days. CD8^{+} T cell proliferation was assessed via CTV dilution. The bar diagram (bottom panel) represents cumulative data from four (F-J) independent experiments. *, $P < 0.05$; **, $P < 0.01$; ***, $P < 0.005$; ****, $P < 0.0001$.

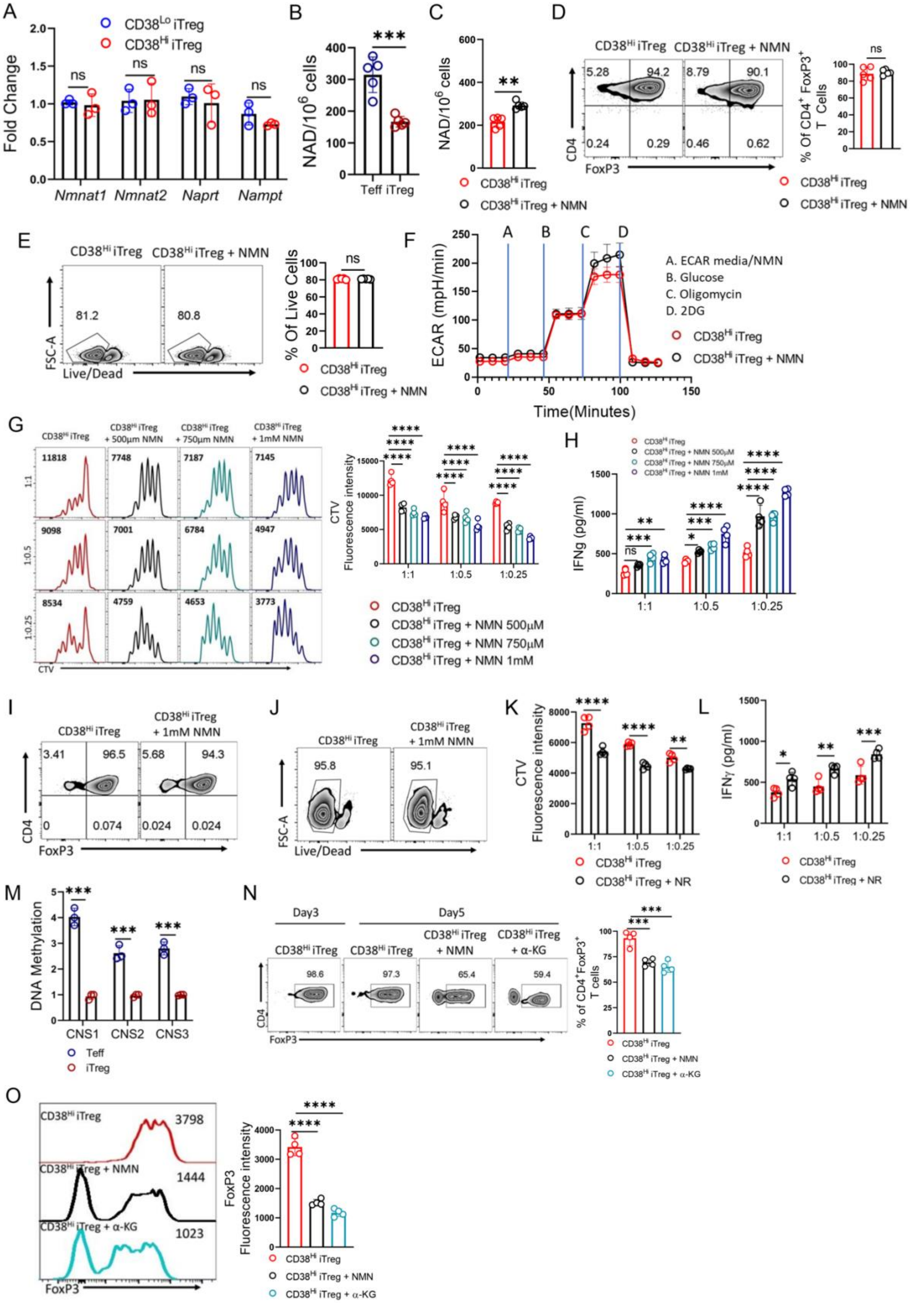

A
CD38Lo iTreg
CD38Hi iTreg
Fold Change
ns
Nmnat1
Nmnat2
Naprt
Nampt
B
NAD/10^6 cells
***
Teff
iTreg
C
NAD/10^6 cells
**
CD38Hi iTreg
CD38Hi iTreg + NMN
D
CD38Hi iTreg
CD38Hi iTreg + NMN
5.28
94.2
0.24
0.29
8.79
90.1
0.46
0.62
CD4
FoxP3
% Of CD4+ FoxP3+ T Cells
ns
E
81.2
80.8
FSC-A
Live/Dead
% Of Live Cells
F
ECAR (mpH/min)
Time(Minutes)
A. ECAR media/NMN
B. Glucose
C. Oligomycin
D. 2DG
G
CD38Hi iTreg + 500µm NMN
CD38Hi iTreg + 750µm NMN
CD38Hi iTreg + 1mM NMN
11818
7748
7187
7145
9098
7001
6784
4947
8534
4759
4653
3773
1:1
1:0.5
1:0.25
CTV
CTV Fluorescence intensity
CD38Hi iTreg + NMN 500µM
CD38Hi iTreg + NMN 750µM
CD38Hi iTreg + NMN 1mM
H
IFNg (pg/ml)
I
CD38Hi iTreg + 1mM NMN
3.41
96.5
0
0.074
5.68
94.3
0.024
0.024
J
95.8
95.1
K
CTV Fluorescence intensity
CD38Hi iTreg + NR
L
IFNγ (pg/ml)
M
DNA Methylation
CNS1
CNS2
CNS3
Teff
iTreg
N
Day3
Day5
CD38Hi iTreg + α-KG
98.6
97.3
65.4
59.4
% of CD4+FoxP3+ T cells
O
3798
1444
1023
FoxP3 Fluorescence intensity

Figure S4. Replenishing cellular NAD^+ pool with NMN mitigates the suppressive function of $CD38^{Hi}$ iTregs. (A) qPCR analysis of genes in FACS-sorted $CD38^{Hi}$ and $CD38^{Lo}$ iTregs. (B-C) Intracellular NAD^+ levels in (B) Teff and iTregs and (C) $CD38^{Hi}$ iTregs cultured overnight in the presence or absence of NMN. (D-F) $CD38^{Hi}$ iTregs cultured overnight in the presence or absence of NMN were assessed for: (D) frequency of $CD4^+FoxP3^+$ cells, (E) cell viability using live/dead staining, and (F) ECAR in response to glucose, oligomycin, and 2DG. (G-H) $CD38^{Hi}$ iTregs cultured overnight with indicated doses of NMN were co-cultured with CTV-labelled naïve T cells in the presence of a T cell activation cocktail (anti-CD3/CD28) for three days and (G) $CD8^+$ T cell proliferation was assessed via CTV dilution. (H) Supernatant from (G) was used to measure IFN-γ levels. (I-J) $CD38^{Hi}$ iTregs cultured overnight in the presence or absence of NMN (1 mM) were assessed for: (I) frequency of $CD4^+FoxP3^+$ cells, and (J) cell viability using live/dead staining. (K-L) $CD38^{Hi}$ iTregs cultured overnight in the presence or absence of NR (400 μM) were co-cultured with CTV-labelled naïve T cells in the presence of a T cell activation cocktail (anti-CD3/CD28) for three days, and (K) $CD8^+$ T cell proliferation was assessed via CTV dilution. (L) Supernatant from (K) was used to measure IFN-γ levels. (M) qPCR-based analysis of CpG methylation at CNS regions of the *Foxp3* locus. (N) Frequency of $CD4^+FoxP3^+$ T cells on day 3 of iTreg differentiation (left) in the $CD38^{Hi}$ iTreg subset and after being cultured for an additional two days following overnight treatment with either NMN or α-KG. (O) The fluorescence intensity of FoxP3 from the experimental groups mentioned in (N). Data are representative of three (A), five (B-E), four (F-L), three (M), and four (N-O) independent experiments. *, $P < 0.05$; **, $P < 0.01$; ***, $P < 0.005$; ****, $P < 0.0001$.

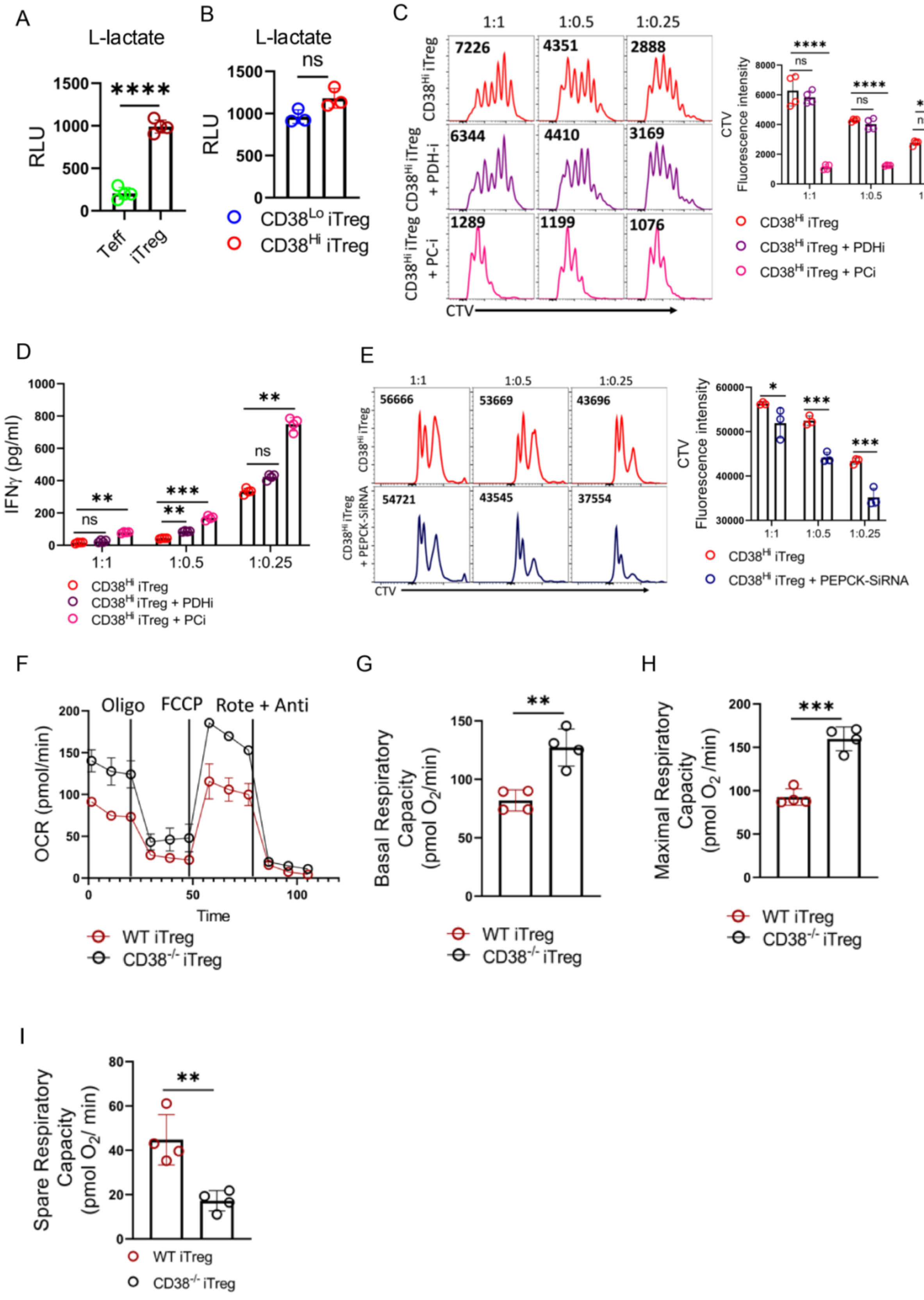

A
L-lactate
RLU
Teff
iTreg
B
L-lactate
ns
CD38Lo iTreg
CD38Hi iTreg
C
1:1
1:0.5
1:0.25
CD38Hi iTreg
CD38Hi iTreg + PDH-i
CD38Hi iTreg + PC-i
7226
4351
2888
6344
4410
3169
1289
1199
1076
CTV
CTV Fluorescence intensity
CD38Hi iTreg + PDHi
CD38Hi iTreg + PCi
D
IFNγ (pg/ml)
E
56666
53669
43696
54721
43545
37554
CD38Hi iTreg + PEPCK-SiRNA
F
Oligo
FCCP
Rote + Anti
OCR (pmol/min)
Time
WT iTreg
CD38-/- iTreg
G
Basal Respiratory Capacity (pmol O2/min)
H
Maximal Respiratory Capacity (pmol O2/min)
I
Spare Respiratory Capacity (pmol O2/ min)

Figure S5. CD38 determines the metabolic routing of lactate in Tregs. (A-B) Determining Intracellular lactate levels in (A) Teff and iTregs, and (B) $CD38^{Hi}$ and $CD38^{Lo}$ iTregs. (C-D) iTregs were differentiated in complete media and sorted into the $CD38^{Hi}$ population. These were then treated overnight with the indicated inhibitors in the same media and were used for co-culturing with CTV-labeled naïve T cells in the presence of a T cell activation cocktail (anti-CD3/CD28) for three days and assessed for (C) $CD8^+$ T cell proliferation via CTV dilution and (D) measuring IFN-γ levels from the supernatant of the experiment shown in (C). (E) T cell proliferation was assessed via CTV dilution after three days of co-culturing naïve T cells with iTregs that were differentiated in glucose-free, L-lactate-containing media, following the indicated RNAi-mediated knockdown. (F-I) WT and $CD38^{-/-}$ iTregs differentiated in glucose-free, L-lactate containing media were assessed for (F) Oxygen consumption rate (OCR) under basal condition and in response to the indicated inhibitors, (G) basal respiratory capacity, (H) maximal respiratory capacity, and (I) spare respiratory capacity. Data are representative of three (A, B, and E) and four (C-D and F-I) independent experiments. *, $P < 0.05$; **, $P < 0.01$; ***, $P < 0.005$; ****, $P < 0.0001$.

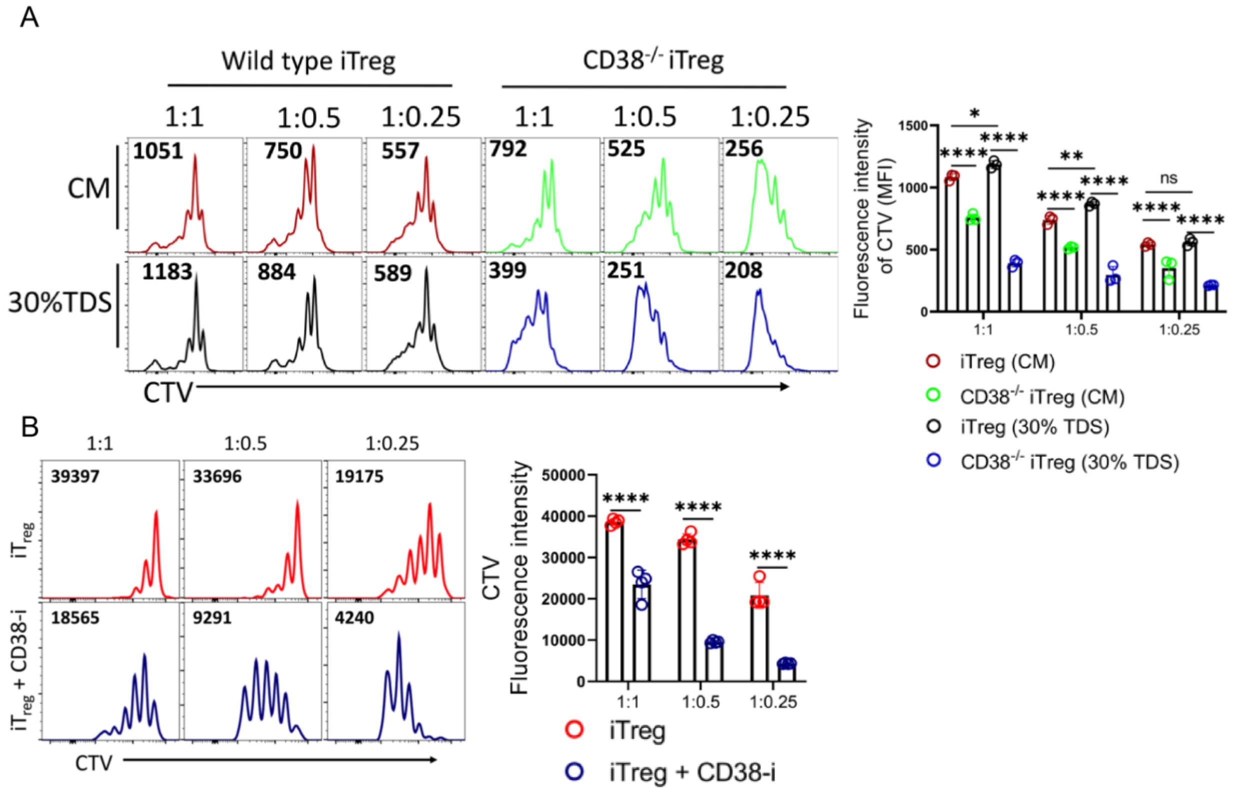
A
Wild type iTreg
CD38-/- iTreg
1:1
1:0.5
1:0.25
CM
30%TDS
1051
750
557
792
525
256
1183
884
589
399
251
208
CTV
Fluorescence intensity of CTV (MFI)
iTreg (CM)
CD38-/- iTreg (CM)
iTreg (30% TDS)
CD38-/- iTreg (30% TDS)
B
39397
33696
19175
18565
9291
4240
iTreg
iTreg + CD38-i
CTV Fluorescence intensity
iTreg
iTreg + CD38-i

Figure S6. **CD38 is crucial for maintaining the functionality of Tregs in nutrient-deprived and hypoxic microenvironments.** (A) WT and $CD38^{-/-}$ iTreg were differentiated either in complete media (CM) or in 30% TDS and were assessed for evaluating suppressive potential by checking the proliferation of CTV-labeled $CD8^+$ T cells from the co-culture. The adjacent bar diagram represents data from three independent experiments. (B) Naïve $CD4^+$ T cells were differentiated into iTregs under hypoxic conditions, with or without CD38i, and their suppressive potential was evaluated by assessing the proliferation of CTV-labeled $CD8^+$ T cells in co-culture. The adjacent bar diagram represents data from four independent experiments. *, $P < 0.05$; **, $P < 0.01$; ***, $P < 0.005$; ****, $P < 0.0001$.

Table S1: Antibodies and cytokines

Antibodies & cytokines	Source	Clone# and Cat#
Anti-mouse CD3	BioXcell	Clone: 145–2C11; Cat# BE001–1
Anti-mouse CD28	BioXcell	Clone: 37.51; Cat# BE0015–1
Recombinant mouse IL2	Peprotech	Cat# 212-12
Recombinant mouse IL12	Peprotech	Cat#210-12
Human TGFβ	Peprotech	Cat# 100-21C
Anti-mouse IFNγ	BioXcell	BE0055
Anti mouse IL4	BioXcell	BE0045
Anti- mouse CD4-PE/Cy7	Thermo Fisher Scientific	Clone: GK1.5; Cat# 25–0041–82
Anti-mouse CD8-AF700	Thermo Fisher Scientific	Clone: 53–6.7; Cat# 56–0081–82
Anti-mouse IFNg-APC	Thermo Fisher Scientific	Clone: XMG1.2; Cat# 17–7311–82
Anti-mouse TNFα-APC/Cy7	Biolegend	Clone: MP6-XT22; Cat# 506344
Anti-mouse GzmB	Biolegend	Clone:QA16A02; Cat# 372208
Anti-mouse CD25-APC	Thermo Fisher Scientific	Clone: PC61.5; Cat# 17–0251–82
Anti-mouse CD8-APC/Cy7	Thermo Fisher Scientific	Clone: 53–6.7; Cat# A15386
Anti-mouse CD4-BUV 395	BD Bioscience	Clone: GK1.5; Cat# 563790
Anti-mouse CD8-Percp/Cy5.5	BD Bioscience	Clone: 53–6.7; Cat# 551162
Anti-mouse CD8-FITC	Biolegend	Clone: 53–6.7; Cat# 100706
Anti mouse CD38-PE	Invitrogen	Clone: 90; Cat# 12-0381-82
Anti-mouse CD38-APC/Cy7	Biolegend	Clone: 90; Cat#102728
Anti-mouse CD38-APC	BD Bioscience	Clone: 90; Cat#562769
Anti-mouse FoxP3-PE/Cy7	Invitrogen	Clone: FJK-16s; Cat#25-5773-82
Anti-mouse FoxP3-APC	Invitrogen	Clone: FJK-16s; Cat#17-5773-82
Anti-mouse Brdu-APC	Biolegend	Clone: 3D4; Cat# 364114
Anti-human Foxp3	Invitrogen	Clone: PCH101; Cat# 25-4776-42
Anti-human CD38	Biolegend	Clone: HIT2; Cat# 303534
Anti-human CD8-AF700	BD Bioscience	Clone: OKT-8; Cat# 557945
Anti-human CD4-BUV395	BD Bioscience	Clone: RPA-T4; Cat#564724

Table S2: Mouse primer sequence

Gene	Forward	Reverse	Source
Glut1	CAGTTCGGCTATAACACTGGTG	GCCCCCGACAGAGAAGATG	IDT, Coralville
HKI	CGGAATGGGGAGCCTTTGG	GCCTTCCTTATCCGTTTCAATGG	IDT, Coralville
HKII	GGAACCGCCTAGAAATCTCC	GGAGCTCAACCAAAACCAAG	IDT, Coralville
Pfk	AGGAGGGCAAAGGAGTGTTT	TTGGCAGAAATCTTGGTTCC	IDT, Coralville
Enol	AAAGATCTCTCTGGCGTGGA	CTTAACGCTCTCCTCGGTGT	IDT, Coralville

Tpi	CCAGGAAGTTCTTCGTTGGGG	CAAAGTCGATGTAAGCGGTGG	IDT, Coralville
Pgam	TCTGTGCAGAAGAGAGCAATCC	CTGTCAGACCGCCATAGTGT	IDT, Coralville
Ctla4	TTTTGTAGCCCTGCTCACTCT	CTGAAGGTTGGGTCACCTGTA	Eurofins, Luxembourg
Icos	TAAAGTGTCCCTGTTTTGTGTCC	ATTGCACCGACTTCAGTCTCT	Eurofins, Luxembourg
Nrp1	GACAAATGTGGCGGGACCATA	TGGATTAGCCATTCACACTTCTC	Eurofins, Luxembourg
IL2ra	AACCATAGTACCCAGTTGTCGG	TCCTAAGCAACGCATATAGACCA	Eurofins, Luxembourg
Ikzf2	CCGTACCTGGTCATCACAGAG	CAGTCTCGAAGCTCGATGGC	Eurofins, Luxembourg
Entpd1	AAGGTGAAGAGATTTTGCTCCAA	TTTGTTCTGGGTCAGTCCCAC	Eurofins, Luxembourg
Nt5e	GGACATTTGACCTCGTCCAAT	GGGCACTCGACACTTGGTG	Eurofins, Luxembourg
Lag3	CTGGGACTGCTTTGGGAAG	GGTTGATGTTGCCAGATAACCC	Eurofins, Luxembourg
B-Actin	ACGTAGCCATCCAGGCTGGTG	TGGCGTGAGGGAGAGCAT	Eurofins, Luxembourg
CNS1	TGGGAGTTAGATTGTTTGGAATAAT	CCTCAAAAAAATCCTCTAAAATAAATACTA	Eurofins, Luxembourg
CNS2	ATTTGAATTGGATATGGTTTGT	AACCTTAAACCCCTCTAACATC	Eurofins, Luxembourg
CNS3	GTTTAGAATGGGGTAAGTAGGGTG	TTACCTAAAACCCATAAACCACTTC	Eurofins, Luxembourg
-1.5 kb	TGTTAGGGTATTAAAGGTTGGAAGTT	CCAATTTTCCTAAAACCAACAATAT	Eurofins, Luxembourg
